# *Dictyostelium discoideum* as a Novel Host System to Study the Interaction between Phagocytes and Yeasts

**DOI:** 10.3389/fmicb.2016.01665

**Published:** 2016-10-21

**Authors:** Barbara Koller, Christin Schramm, Susann Siebert, János Triebel, Eric Deland, Anna M. Pfefferkorn, Volker Rickerts, Sascha Thewes

**Affiliations:** ^1^Department of Biology, Chemistry, Pharmacy, Institute for Biology – Microbiology, Freie Universität BerlinBerlin, Germany; ^2^FG16, Robert Koch InstituteBerlin, Germany

**Keywords:** host-pathogen interaction, *Saccharomyces*, *Candida*, phagocytosis, virulence, autophagy, amoebae

## Abstract

The social amoeba *Dictyostelium discoideum* is a well-established model organism to study the interaction between bacteria and phagocytes. In contrast, research using *D. discoideum* as a host model for fungi is rare. We describe a comprehensive study, which uses *D. discoideum* as a host model system to investigate the interaction with apathogenic (*Saccharomyces cerevisiae*) and pathogenic (*Candida* sp.) yeast. We show that *Dictyostelium* can be co-cultivated with yeasts on solid media, offering a convenient test to study the interaction between fungi and phagocytes. We demonstrate that a number of *D. discoideum* mutants increase (*atg1*^−^, *kil1*^−^, *kil2*^−^) or decrease (*atg6*^−^) the ability of the amoebae to predate yeast cells. On the yeast side, growth characteristics, reduced phagocytosis rate, as well as known virulence factors of *C. albicans* (*EFG1, CPH1, HGC1, ICL1*) contribute to the resistance of yeast cells against predation by the amoebae. Investigating haploid *C. albicans* strains, we suggest using the amoebae plate test for screening purposes after random mutagenesis. Finally, we discuss the potential of our adapted amoebae plate test to use *D. discoideum* for risk assessment of yeast strains.

## Introduction

Since their first description in 1869 (Brefeld, 1869) social amoebae have become a model organism for several aspects, ranging from cell biology and developmental biology to molecular medicine (Müller-Taubenberger et al., 2013). Accordingly, the most-investigated species of the social amoebae, *Dictyostelium discoideum*, is one of the non-mammalian model organisms for biomedical research, as defined by the National Institutes of Health (USA; http://www.nih.gov/science/models/). In the last 15 years *D. discoideum* has also been established as a host model to study the interaction with intracellular pathogenic bacteria like *Legionella pneumophila, Mycobacterium marinum*, and *M. tuberculosis, Salmonella typhimurium, Pseudomonas aeruginosa*, and others (for reviews see Clarke, 2010; Bozzaro and Eichinger, 2011). Subsequently, protocols have been established to study *Dictyostelium*-bacteria interactions (Froquet et al., 2009; Bozzaro, 2013; Tosetti et al., 2014).

However, to date, only few publications have investigated the interaction between *Dictyostelium* and fungi. It has been shown that co-incubation of *D. discoideum* with *Cryptococcus neoformans* enhances the virulence of the fungus in a mouse model of infection (Steenbergen et al., 2003), which might be explained by a capsule enlargement of *C. neoformans* during the co-incubation (Chrisman et al., 2011). Recently, the interaction of the filamentous fungus *Aspergillus fumigatus* with *D. discoideum* has been investigated (Hillmann et al., 2015). The authors found that known virulence determinants of *A. fumigatus* also protect against predation by *Dictyostelium*.

Research on the interaction between *D. discoideum* and yeasts is mainly focused on the investigation of phagocytosis processes of the amoeba (Rivero and Maniak, 2006). One reason why the interaction of amoebae and fungi is only poorly investigated might be the fact that protocols used to study bacteria failed when used for the investigation of fungi (Froquet et al., 2009). Nevertheless, since the genome of *D. discoideum* is fully sequenced (Eichinger et al., 2005) and *D. discoideum* is easy to cultivate and highly amenable to genetic manipulations (Fey et al., 2007; Gaudet et al., 2007), this amoebae would be an ideal phagocytic host to study interaction with yeasts. Therefore, we successfully adapted a protocol originally used to study the interaction between *Acanthamoeba castellanii* and *L. pneumophila* (Albers et al., 2005) for use with *Dictyostelium* and apathogenic yeasts (*Saccharomyces cerevisiae*) and the pathogenic yeasts *Candida albicans* and *C. glabrata*. We show that different yeast strains have differences in their resistance toward predation by *D. discoideum*. Pathogenic yeasts are usually resistant against predation. Finally, we investigated host factors as well as fungal factors that might have an influence on the interaction, and found that autophagy plays a crucial role in the part of the host *D. discoideum*.

## Materials and methods

### Strains and growth conditions of dictyostelia

All *D. discoideum* strains used in this study are listed in Table 1. Strains were grown axenically in HL5 medium without glucose (ForMedium, Norfolk, UK) supplemented with 1.8% (w/v) maltose (in the following referred to as “HL5”) and 100 μg/ml ampicillin. For transgenic cell lines the medium was additionally supplemented with appropriate antibiotics (20 μg/ml G418 or 5 μg/ml blasticidin). Cells were incubated on a rotary shaker at 22°C and 150 rpm in the dark. Cell numbers were determined using a Neubauer counting chamber. For non-axenic growth, *E. coli* B/r (Witkin, 1946) was grown overnight at 37°C and 200 rpm in LB-medium (10 g/l tryptone, 5 g/l yeast extract, 10 g/l NaCl). The next day, the bacteria were washed twice with ice-cold SP-buffer (15 mM KH_2_PO_4_, 2 mM Na_2_HPO_4_, pH 6.0) and cells were adjusted to OD_600_ = 6 in SP-buffer. Social amoebae listed in Table 2 were inoculated in the *E. coli* B/r suspension and incubated on a rotary shaker with 150 rpm at 22°C in the dark. For co-incubation studies with yeast, amoebae were washed free of bacteria twice with ice-cold SP-buffer by differential centrifugation at 800 g and were re-suspended in SP-buffer.

**Table 1:** ***Dictyostelium discoideum* strains used in this study**.

**Strain**	**Genotype**	**Parental strain**	**References**
AX2	axeA2, axeB2, axeC2	NC4	Watts and Ashworth, 1970
DH1	axeA1, axeB1, axeC1, pyr5-6-[pRG24], ura^−^	NC4	Caterina et al., 1994
*atg1^−^*	axeA1, axeB1, axeC1, atg1-1[pAtg1-1], bsR	DH1	Otto et al., 2004
*atg5^−^*	axeA1, axeB1, axeC1, pyr5-6-[pRG24], ura-, atg5-[atg5-KO], bsR	DH1	Otto et al., 2003
*atg6^−^*	axeA1, axeB1, axeC1, pyr5-6-[pRG24], ura-, atg6-[atg6-HR], bsR	DH1	Otto et al., 2004
*atg7^−^*	axeA1, axeB1, axeC1, pyr5-6-[pRG24], ura-, atg7-[atg7-KO], bsR	DH1	Otto et al., 2003
*atg8^−^*	axeA1, axeB1, axeC1, pyr5-6-[pRG24], ura-, atg8-[atg8-KO], bsR	DH1	Otto et al., 2004
CNA-RNAi	axeA2, axeB2, axeC2, [canA-RNAi], neoR	AX2	Thewes et al., 2014
CNB-RNAi	axeA2, axeB2, axeC2, [cnbA-RNAi], neoR	AX2	Boeckeler et al., 2006
*dymA^−^*	axeA2, axeB2, axeC2, dymA-, bsR	AX2	Wienke et al., 1999
*kil1^−^*	axeA1, axeB1, axeC1, pyr5-6-[pRG24], ura-, kil1-[kil1-bsr], bsR	DH1	Benghezal et al., 2006
*kil2^−^*	axeA1, axeB1, axeC1, pyr5-6- [pRG24], ura-, kil2-, [kil2-bsr], bsr	DH1	Lelong et al., 2011
*phg1A*^−^	axeA1, axeB1, axeC1, pyr5-6-[pRG24], ura-, phg1a-[pPHG1a], bsR	DH1	Cornillon et al., 2000
*racH^−^*	axeA2, axeB2, axeC2, racH::BSR	AX2	Somesh et al., 2006
*rdeA*^−^	axeA1, axeB1, axeC1, pyr5-6-[pRG24], rdeA-[pDIV5], ura+	DH1	Chang et al., 1998
*sibA^−^*	axeA1, axeB1, axeC1, pyr5-6-[pRG24), sibA-, ura+	DH1	Cornillon et al., 2006
*wshA^−^*	axeA2, axeB2, axeC2, wshA-[wshA::bsr], bsR	AX2	Carnell et al., 2011

All strains were grown axenically.

**Table 2:** **Non-axenic social amoebae used in this study**.

**Species**	**Strain**	**References**
*Dictyostelium caveatum*	BW1 (ATCC66413)	ATCC
*Dictyostelium polycephalum*	natural isolate	T. Winckler (Jena, Germany)
*Dictyostelium purpureum*	natural isolate	T. Winckler (Jena, Germany)
*Polysphondylium pallidum*	PN500	Francis, 1975

All strains were grown in association with Escherichia coli B/r.

### Fungal strains and growth conditions

All *S. cerevisiae* and *Candida* strains used in this study are listed in Tables 3, 4. Yeast cells were grown in YPD-medium (10 g/l yeast extract, 10 g/l peptone, 20 g/l glucose) at 30°C and 180 rpm on a rotary shaker. For the induction of *flo1*-expression in strain KV210, galactose [2% (w/v)] was added to the YPD-medium. YPD-plates contained 2% (w/v) agar. For flocculation analysis, yeast cells were grown overnight in YPD as described above. Five milliliter of the grown cultures were transferred to test tubes, carefully mixed, and incubated for 30 min without moving at room temperature. Sedimentation (= flocculation) of cells was monitored with a digital camera. Growth of yeasts in different media was determined photometrically at 600 nm. Yeasts were grown either in YPD, HL5 without glucose supplemented with maltose (“HL5,”) or HL5 without glucose and maltose (“AXoM”) at 30°C. OD_600_ was determined at the indicated time points.

**Table 3:** ***Saccharomyces cerevisiae* strains used in this study**.

**Strain**	**Genotype**	**References**
RKI 05-0082-01	Wild type (bloodstream isolate)	Robert Koch Institute, Berlin, Germany
RKI 05-0082-02	Wild type (bloodstream isolate)	Robert Koch Institute, Berlin, Germany
RKI 07-0060	Wild type (bloodstream isolate)	Robert Koch Institute, Berlin, Germany
RKI 07-0061	Wild type (bloodstream isolate)	Robert Koch Institute, Berlin, Germany
Baker's yeast	Wild type (bakery isolate)	Local bakery, Berlin, Germany
Brewer's yeast	Wild type (brewery isolate)	Versuchs- und Lehranstalt für Brauerei, Berlin, Germany
BY25558	*MATa ura3Δ0 URA3-TDH3p-FLO1*	Nonklang et al., 2009
BY25559	*MATa ura3Δ0 URA3-TDH3p-FLO5*	Nonklang et al., 2009
BY25560	*MATa ura3Δ0 URA3-TDH3p-FLO9*	Nonklang et al., 2009
BY25561	*MATa ura3Δ0 URA3-TDH3p-FLO10*	Nonklang et al., 2009
BY4741	*MATa his3Δ*1 *leu2Δ*0 *lys2Δ*0 *ura3Δ*0	Purevdorj-Gage et al., 2007
INVSc1	*MATa his3Δ1 leu2 trp1-289 ura3-52*	Life Technologies, Darmstadt, Germany
KV210	*MATa his3Δ*1 *leu2Δ*0 *lys2Δ*0 *ura3Δ*0 *Gal*p*-FLO1*	Smukalla et al., 2008
KV22	*MATa his3Δ*1 *leu2Δ*0 *lys2Δ*0 *ura3Δ*0 *flo1::KANMX*	Smukalla et al., 2008
KV84	Wildtype (natural flocculation)	Beauvais et al., 2009
TH2-1B	*MATa SUC2 mal mel gal2 CUP1 mnn1 mnn2*	Clarke, 2010
w303a	*MATa leu2-3,112 trp1-1 can1-100 ura3-1 ade2-1 his3-11,15*	Wallis et al., 1989
w303a/α	*MATa/*α *leu2-3,112 trp1-1 can1-100 ura3-1 ade2-1 his3-11,15*	Wallis et al., 1989
w303α	*MATα leu2-3,112 trp1-1 can1-100 ura3-1 ade2-1 his3-11,15*	Wallis et al., 1989
Y-187	*MATa gal4 gal80 his3 trpl-901 ade2-101 ura3-52 leu2-3,-112 URA3 GAL*->*lacZ met^−^*	Harper et al., 1993
Y-190	*MATa leu2-3,112 ura3-52 trp1-901 his3-Δ200 ade2-101 gal4Δ gal80Δ URA3 GAL-lacZ, LYS GAL-HIS3, cyh*	Harper et al., 1993

**Table 4:** ***Candida* strains used in this study**.

**Strain**	**Genotype**	**Parental strain**	**References**
***Candida albicans***			
**Diploid**			
SC5314	Wild type	–	Gillum et al., 1984
ATCC10231	Wild type	–	Kretschmar et al., 1999; Thewes et al., 2008
CAI-4 + CIp10	Δ*ura3*::*imm434*/Δ*ura3*::*imm434* + CIp10	SC5314	Fonzi and Irwin, 1993; Murad et al., 2000
Δ*cph1*	Δ*ura3*::*imm434*/Δ*ura3*::*imm434* Δ*cph1*::*hisG*/Δ*cph1*::*hisG*	CAI-4	Liu et al., 1994
Δ*cph1/*Δ*efg1*	Δ*ura3*::*imm434*/Δ*ura3*::*imm434* Δ*cph1*::*hisG*/Δ*cph1*::*hisG* Δ*efg1*::*hisG*/Δ*efg1*::*hisG* + CIp10	CAI-4	Lo et al., 1997
Δ*dfg16*	Δ*ura3*::*imm434*/Δ*ura3*::*imm434* Δ*dfg16*::*hisG*/Δ*dfg16*::*hisG* + CIp10	CAI-4	Thewes et al., 2007
Δ*efg1*	Δ*ura3*::*imm434*/Δura3::imm434 Δ*efg1*::*hisG*/Δ*efg1*::*hisG* + CIp10	CAI-4	Lo et al., 1997
Δ*hgc1*	Δ*ura3*::*imm434*/Δ*ura3*::*imm434* Δ*his1*::*hisG*/Δ*his1*::*hisG* Δ*arg4*::*hisG*/Δ*arg4*::*hisG* Δ*hgc1*::*ARG4*/Δ*hgc1*::*HIS1* + CIp10	BWP17	Zheng et al., 2004
Δ*icl1*	Δ*ura3*::*imm434*/Δ*ura3*::*imm434* Δ*icl1*::*hisG*/Δ*icl1*::*hisG* + CIp10	CAI-4	Ramírez and Lorenz, 2007
Δ*sap1-3*	Δ*ura3*::*imm434*/Δ*ura3*::*imm434* Δ*sap1*::*hisG*/Δ*sap1*::*hisG* Δ*sap2*::*hisG*/Δ*sap2*::*hisG* Δ*sap3*::*hisG*/Δ*sap3*::*hisG* + CIp10	CAI-4	Kretschmar et al., 2002
Δ*sap4-6*	Δ*ura3*::*imm434*/Δ*ura3*::*imm434* Δ*sap4*::*hisG*/Δ*sap4*::*hisG* Δ*sap5*::*hisG*/Δ*sap5*::*hisG* Δ*sap6*::*hisG*/Δ*sap6*::*hisG* + CIp10	CAI-4	Sanglard et al., 1997
**Haploid**			
GZY805 (control)	*MTLa his4* Δ*ura3::HIS4*	GZY803	Hickman et al., 2013
GZY806 (Δ*hgc1*)	*MTLa his4* Δ*ura3::HIS4* Δ*hgc1::UFP*	GZY805	Hickman et al., 2013
GZY822 (control)	*MTLa his4* Δ*ura3::HIS4* Δ*arg4::FRT* Δ*his1::FRT*	GZY815	Hickman et al., 2013
GZY824 (Δ*sla1*)	*MTLa his4* Δ*ura3::HIS4* Δ*arg4::FRT* Δ*his1::FRT* Δ*sla1::UFP*	GZY822	Hickman et al., 2013
GZY825 (Δ*ace2*)	*MTLa his4* Δ*ura3::HIS4* Δ*arg4::FRT* Δ*his1::FRT* Δ*ace2::ARG4*	GZY822	Hickman et al., 2013
GZY833 (Δ*sec3*)	*MTLa his4* Δ*ura3::HIS4* Δ*arg4::FRT* Δ*his1::FRT* Δ*sec3::UFP*	GZY822	Hickman et al., 2013
GZY834 (Δ*rvs167*)	*MTLa his4* Δ*ura3::HIS4* Δ*arg4::FRT* Δ*his1::FRT* Δ*rvs167::ARG4*	GZY822	Hickman et al., 2013
***Candida glabrata***			
CBS138/ATCC2001	Wild type	–	CBS/ATCC
***Candida krusei***			
ATCC6258	Wild type	–	ATCC

### Amoebae plate test

**Figure 1:**
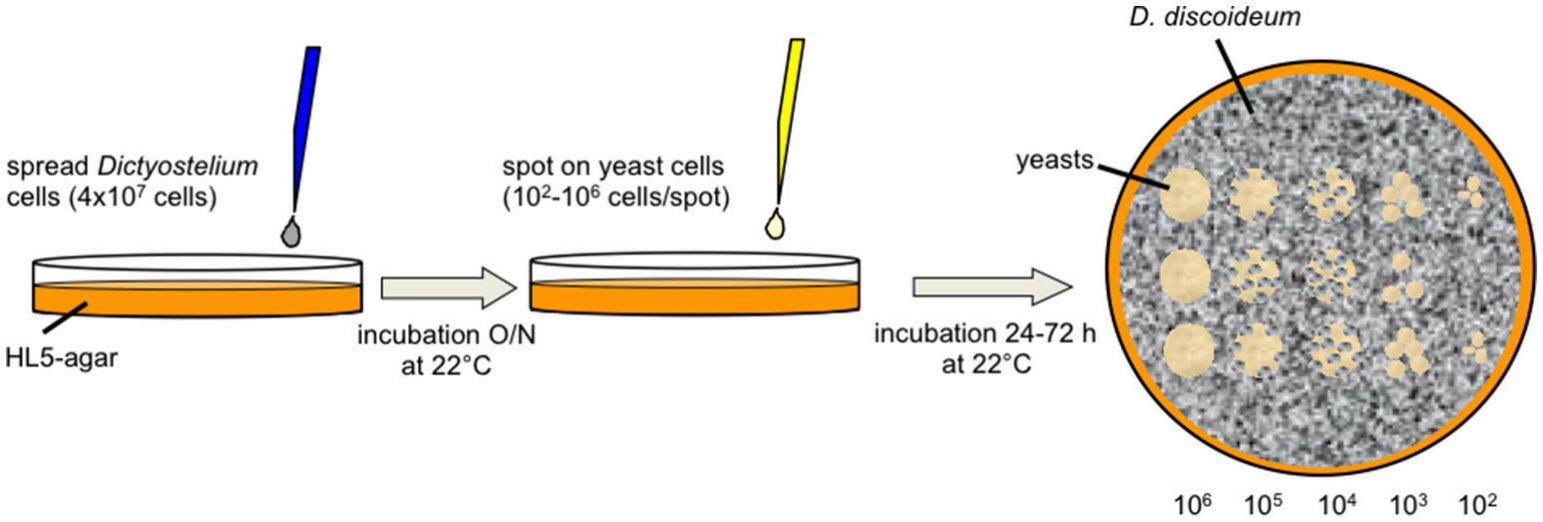
**Diagram of the amoebae plate test**. *D. discoideum* cells were spread on HL5-agar. After incubation overnight, serial dilutions of yeast cells were spotted on the surface. Co-incubation of amoebae and yeast cells was observed up to 72 h after spotting the yeasts.

The amoebae plate test (Figure 1) was adapted from Albers et al. (2005). Amoebae were grown to mid-logarithmic phase (4–8 × 10^6^ cells/ml), washed once with SP-buffer and adjusted to 8 × 10^6^ cells/ml in SP. Five milliliter of the suspension were spread on solid HL5 agar [2% (w/v)] in ø9 cm petri dishes. After 30 min the supernatant was aspirated and plates were incubated overnight at 22°C. To test non-axenically grown amoebae, HL5 agar plates were supplemented with ampicillin (100 μg/ml) and streptomycin (50 μg/ml) to prevent growth of residual bacteria. The next day, yeast cells grown overnight in YPD at 30°C were washed twice with SP, counted, and adjusted to 2 × 10^8^ cells/ml in SP. Flocculating yeasts were additionally washed before counting in 250 mM EDTA to dissociate cells from flocs. The yeast suspensions were serially diluted 1:10 in SP (2 × 10^7^–2 × 10^4^ cells/ml) and 5 μl of each dilution were spotted on the amoeba plates and on plates without amoebae (control), respectively. Control plates for non-axenically grown amoebae were supplemented with ampicillin and streptomycin. Plates were incubated at 22°C. Documentation was done at the indicated time points. Amoebae plate tests were repeated at least three times (biological replicates) per strain.

### Quantitative co-incubation

To quantify the effects of the co-incubation of *Dictyostelium* with yeast cells, amoebae from mid-logarithmic growth phase were washed twice with ice-cold SP and adjusted to 3.3 × 10^5^ cells/ml in AXoM. Amoebae were seeded in flat-bottomed 96-well plates (150 μl/well). Yeast cells were grown overnight in YPD, washed twice in SP, and adjusted to 2 × 10^6^ cells/ml in SP. Serial 1:4 dilutions were prepared in SP down to 1.95 × 10^3^ cells/ml. Fifty microliters of each dilution were added to the amoebae in triplicate, resulting in multiplicity of infections (MOI) of 2, 0.5, 0.125, 0.03125, 0.00781, 0.00195, respectively. As a control, yeast cells were inoculated in triplicate in 150 μl AXoM without amoebae. Plates were incubated at 22°C. At the indicated time points amoebae were lysed by adding 5 μl Triton X-100 (20%) and yeast cells were dissociated by adding 50 μl EDTA (250 mM). After appropriate dilution, yeast cells were spread on YPD-plates, incubated at 30°C, and colony-forming units (cfu) were counted. Triton X-100 and EDTA had no effect on the survival of yeast cells in our assay (data not shown). Co-incubation was repeated for each strain three times (biological replicates) including three technical replicates.

### Trypan blue exclusion assay

Survival of the amoebae was investigated using the trypan blue exclusion assay. *D. discoideum* AX2 was grown in HL5 at 22°C to mid-log phase, washed twice with SP-buffer and 900 μl of cells in SP were seeded in triplicate with a density of 1.3 × 10^5^ cells/ml in 24-well plates containing sterile, acid-washed glass coverslips (ø14 mm). Yeast cells were grown overnight at 30°C in YPD, washed twice with SP and 100 μl yeast suspension in SP (2.4 × 10^6^ cells/ml) was added to the amoebae to a final MOI of 2. Plates were incubated at 22°C. At the indicated time points the coverslips were carefully removed from the wells and adhering cells were stained with trypan blue (1% in SP) and directly analyzed under the microscope. Blue *D. discoideum* cells were counted as dead cells, whereas unstained cells were counted as living cells. At least 50 cells were counted per well. As a control *D. discoideum* cells were incubated in SP-buffer without yeast. Experiments were repeated three times (biological replicates).

### Phagocytosis assay

Uptake of yeast cells by the amoebae was quantified microscopically. Yeast strains were grown overnight at 30°C in YPD, washed twice with SP-buffer, and re-suspended in SP-buffer at 1 × 10^8^ cells/ml. *D. discoideum* strains were grown to mid-log phase in HL5 at 22°C, washed twice in SP buffer, and re-suspended in 10 ml AXoM in a 100 ml Erlenmeyer-flask at 1 × 10^6^ cells/ml. Three hundred microliters of the yeast suspension were added to reach an MOI of 3. The preparations were incubated at 22°C on a rotary shaker at 70 rpm. At the indicated time points 1 ml of the yeast/amoeba mixture was withdrawn from the flask, washed twice with SP-buffer and re-suspended in 500 μl SP containing 0.01% calcofluor white. Cells were incubated for 5 min at room temperature, washed once with SP buffer and re-suspended again in 500 μl SP. Phagocytized yeast cells were distinguished from non-phagocytized yeast cells due to the co-localization with amoebae with simultaneous absence of calcufluor staining. Only non-phagocytized cells showed fluorescence using a DAPI-filter (excitation wavelength 460 nm) with the Nikon ECLIPSE 90i fluorescence microscope (Nikon, Düsseldorf, Germany). Uptake of yeast cells by amoebae was calculated according to McKenzie et al. (2010). Phagocytosis experiments were repeated for each strain three times (biological replicates).

### Time-lapse microscopy

The interaction of yeast cells and amoebae was documented for 4–5 h in submerged culture using time-lapse microscopy. *D. discoideum* AX2 cells grown to mid-logarithmic phase in HL5 were washed once with ice-cold SP-buffer and cell pellets were re-suspended in AXoM at a density of 1.3 × 10^6^ cells/ml. Five hundred microliters were spread in 24-well plates. Yeast cells were grown overnight in YPD, washed twice with SP-buffer, and cells were re-suspended in AXoM at a density of 2 × 10^7^ cells/ml. One hundred microliters of the yeast cell suspension were added to the amoebae in 24-well plates to reach an MOI of 3. Interaction of cells was observed at 22°C using the Axiovert 25 microscope with a 20x objective (Carl Zeiss, Jena, Germany). The microscope was equipped with a TCA-3.0 camera (Tuscen Photonics, Fuzhou, China) and pictures were taken using the Micam 2.0 software (Marie van Westen, Groningen, Netherlands; http://www.science4all.nl) with the following settings: capture interval 1 min, total number of frames 240–300, playback 10 frames/s without compression.

### Analysis of hyphae formation

To test for the ability of *C. albicans* strains to form hyphae during co-incubation with the amoebae, mid-log *D. discoideum* AX2 cells grown in HL5 were washed twice with SP buffer and were re-suspended in AXoM at 3.3 × 10^5^ cells/ml. One hundred and fifty microliters of the *Dictyostelium* suspension was applied to 96-well plates and 50 μl of SP-washed yeast cells (grown in YPD overnight at 30°C) were added with an MOI of 0.125. Plates were incubated at 22°C in the dark and pictures were taken at the indicated time points using the Axioskop 2 microscope (Zeiss, Jena, Germany).

To test whether cell-cell-contact is necessary for hyphae formation, amoebae, and yeast cells were separated using Transwell® inserts (HTS Transwell®-24, PET membrane, pore size 0.4 μm; Corning Incorporation, New York, USA). *C. albicans* strains were grown overnight in YPD at 30°C and washed twice with SP buffer. Washed cells were re-suspended in AXoM at 1.7 × 10^6^ cells/ml. Cells (600 μl) were seeded into 24-well plates (lower compartment). Transwell® inserts (upper compartment) were inserted and filled with 100 μl of *D. discoideum* cells (6.8 × 10^6^ cells/ml in AXoM), 100 μl *D. discoideum* cells together with 10^5^ *C. albicans* cells, or 100 μl of *D. discoideum* cells lysed with 25 μl Triton X-100 (20%), respectively. As a control, *C. albicans* cells were treated with 25 μl Triton X-100. Plates were incubated at 22°C and pictures were taken after 48 h.

Hyphae formation was further investigated with culture supernatant and treated lysate. Culture supernatant was taken from stationary phase *D. discoideum* cell cultures (1–2 × 10^7^ cells/ml) after centrifugation at 400 g for 5 min. Cell lysate was prepared by lysing *D. discoideum* cells from mid-log phase with Triton X-100 (see above). Lysate was incubated for 1 h at 4°C or −20°C, or it was boiled for 5 min at 95°C. Additionally, cell lysate was centrifuged at room temperature at 500 g and 5000 g, respectively. One hundred microliters of the supernatant or lysate were added to 1 × 10^5^ *C. albicans* cells in 24-well plates. Plates were incubated at 22°C and pictures were taken after 48 h. All experiments were repeated three times (biological replicates).

### Propidium iodide staining and flow cytometry

To test whether the haploid *C. albicans* strains are still haploid, cells were stained with propidium iodide (PI) and analyzed by flow cytometry. Yeast cells were grown to mid-logarithmic growth phase at 30°C and 5 × 10^6^ cells were centrifuged at 2000 rpm for 5 min. The supernatant was discarded and the cell pellet was fixed in 1 ml ice-cold 70% ethanol. One hundred and fifty microliters of fixed cells (approximately 2–3 × 10^6^ cells) were mixed with 1.5 ml 50 mM sodium citrate. Cells were centrifuged again at 2000 rpm for 5 min and the cell pellet was re-suspended in 250 μl 50 mM sodium citrate containing 0.1 mg/ml RNAse A. The cells were incubated for 2 h at 37°C. After RNAse-treatment, 250 μl 50 mM sodium citrate containing 8 μg/ml PI were added. Cells were separated with a 40 μm Cell Strainer (Biologix, Jinan, China), and immediately analyzed with the CytoFLEX S flow cytometer (excitation wavelength 488 nm, bandpass filters with 585/42 nm and 690/50 nm width; Beckman Coulter, Krefeld, Germany). At least 20,000 cells per sample were analyzed using FlowJo v9.4.11 (FlowJo, LLC, Ashland, OR, USA).

### Image processing

If necessary, images were processed using ImageJ 1.46r (National Institutes of Health, USA). Images were auto-adjusted for brightness and contrast.

## Results

### Establishment of the amoebae plate test

In order to find protocols for studying the interaction of amoebae with yeast cells we also tried to establish the commonly used plaque-assay with different media (Figure S1). Although *Dictyostelium* was able to produce plaques on yeast lawns on different media when spotted on freshly-spread yeast cells, the plaques were not increasing in size, indicating that yeast cells are not the preferred food source for the amoebae. We also investigated the ability of *D. discoideum* to move chemotactically in the direction of yeast cells using an under-agarose chemotaxis assay (Woznica and Knecht, 2006) and discovered that the amoebae were not able to move toward the yeasts (data not shown).

**Figure 2:**
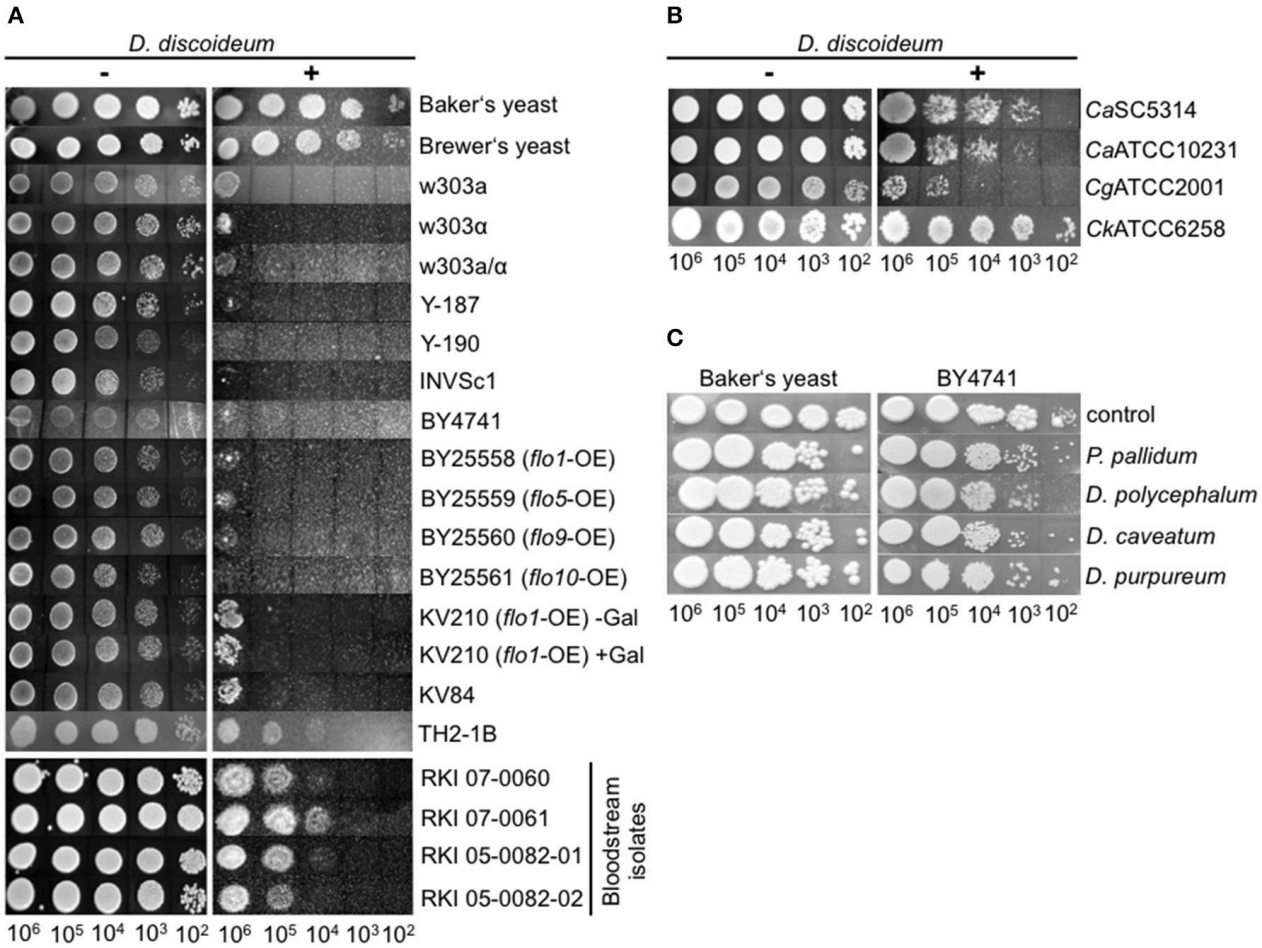
**Growth of different yeast strains in the amoebae plate test. (A)** Different *S. cerevisiae* strains show different growth characteristics during co-incubation with *D. discoideum* AX2. Baker's and Brewer's yeast grew very well, whereas all laboratory strains tested, as well as flocculating strains, grew only poorly. Bloodstream isolates of *S. cerevisiae* showed intermediate growth. **(B)** Pathogenic *Candida* species grew very well on plates containing *D. discoideum*. **(C)** Non-axenic social amoebae could not reduce yeast growth, independently of the strain used. For better visualization, results were assembled from different plates in all plate tests. All plate tests were repeated at least three times by different people.

We therefore looked for alternative protocols to investigate the interaction of amoebae with yeast and adapted the amoebae plate test of Albers et al. (2005). Originally, this test was established to investigate the interaction of *Acanthamoeba* with *Legionella* bacteria. For our purpose we spread *D. discoideum* cells on HL5-agar, spotted serially dilutions of yeast suspensions on the lawn of amoebae, and observed the growth of the yeast up to 72 h (Figure 1). It must be mentioned that the amoebae started to produce fruiting bodies between 24 and 48 h after the spotting of the yeast cells (48–72 h after spreading of the amoebae). This indicates, again, that the yeast cells were not the preferred food source, leading to starvation and subsequent initiation of the developmental cycle of *D. discoideum*. Additionally, *Dictyostelium* seemed to not be able to obtain sufficient nutrients from solidified HL5 medium over a longer time period (more than 48 h; the initiation of the social cycle was not observed during the first 24–48 h after spreading the amoebae). However, we hypothesized that co-incubation of yeasts with amoebae impairs growth of yeasts and tested first several typical laboratory strains (w303a, w303α, w303a/α, Y-187, Y-190, INVSc1, BY4741), “wild type” isolates like Baker's and Brewer's yeast, flocculating strains (BY25558-25561, KV210, KV84; Figure S2), a strain with a cell division defect (TH2-1B), as well as bloodstream isolates (RKI 07-0060, RKI 07-0061, RKI 05-0082-01, RKI 05-0082-02) in combination with the *D. discoideum* axenic strain AX2 (Figure 2A; Tables 1, 3). As demonstrated in Figure 2A, Baker's and Brewer's yeast grew very well on plates containing amoebae, whereas all (non-flocculating) laboratory strains as well as the flocculating strains were inhibited in growth. Otherwise, strain TH2-1B, which has a cell division defect (Clarke, 2010), and the bloodstream isolates showed intermediate growth on amoebae-containing plates (Figure 2A). The result of the “virulent” *S. cerevisiae* bloodstream isolates led us to test secondly the interaction of the amoebae with the pathogenic yeasts, *C. albicans, C. glabrata*, and *C. krusei* (Figure 2B; Table 4). *C. albicans* and *C. glabrata* are the two most common *Candida*-species causing candidemia, whereas *C. krusei* is rarely found (Guinea, 2014). The two investigated filamentous *C. albicans* strains SC5314 and ATCC10231 showed very good growth on the amoebae-plates, with slight differences between the highly virulent SC5314 and the less virulent ATCC10231 (Figure 2B; Thewes et al., 2008). *C. glabrata* is non-filamentous and was comparable in its growth to the *S. cerevisiae* bloodstream isolates. Interestingly, *C. krusei*, which can form only pseudohyphae, was found to grow very well on plates with *Dictyostelium* (Figure 2B).

Subsequently, the ability of other Dictyostelia to interact with yeast cells was tested using species from different taxonomic divisions (Schaap et al., 2006): *D*. *discoideum* and *D. purpureum* from group 4, *D. caveatum* from group 3, *P. pallidum* from group 2, and the group 2/group 3-intermediate species *D. polycephalum* (Figure 2C; Table 2). Except for the *D. discoideum* strains, all other Dictyostelia were non-axenic and had to be grown in association with bacteria. In the amoebae plate test the yeast strain BY4741 as well as the Baker's yeast grew very well in association with non-axenic Dictyostelia (Figure 2C). Therefore, we decided to work in the following experiments solely with axenic *D. discoideum* strains.

### Analysis of yeast factors affecting growth on amoebae plates

**Figure 3:**
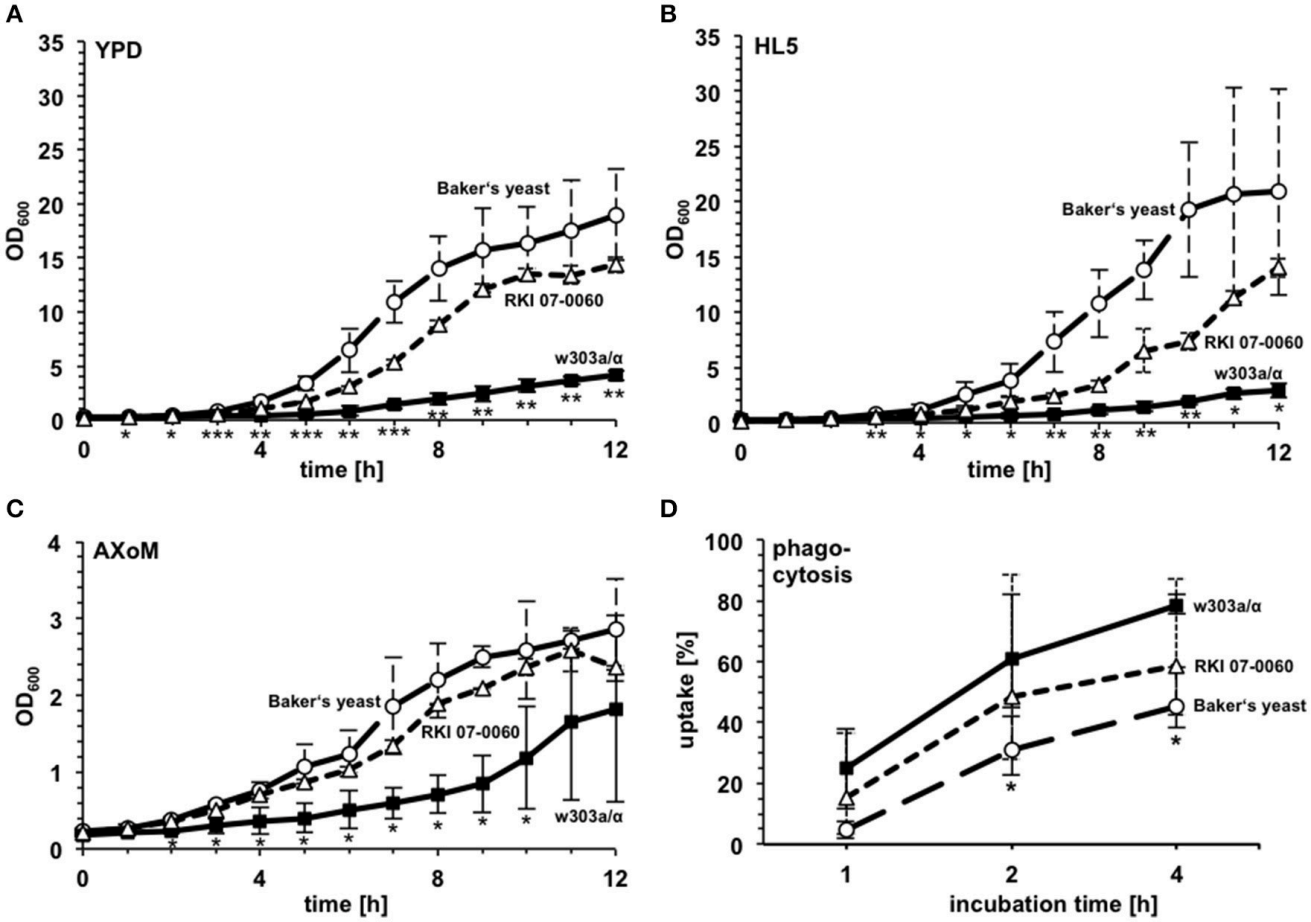
**Growth characteristics and phagocytosis rate of different *S. cerevisiae* strains**. Growth of the Baker's yeast, the laboratory strain w303a/α, and the patient isolate RKI 07-0060 was monitored in YPD **(A)**, HL5 **(B)**, and AXoM **(C)**. ^*^significant difference between Baker's yeast and w303a/α with *P* < 0.05; ^**^significant difference between Baker's yeast and w303a/α, and between RKI 07-0060 and w303a/α, resp., with *P* < 0.05; ^***^significant difference between Baker's yeast and w303a/α, between RKI 07-0060 and w303a/α, and between Baker's yeast and RKI 07-0060, resp., with *P* < 0.05. **(D)** Phagocytosis rate of different *S. cerevisiae* strains during co-incubation with *D. discoideum* AX2. ^*^significant difference between Baker's yeast and w303a/α with *P* < 0.05. Differences between RKI 07-0060 and Baker's yeast were non-significant. All data are represented as mean ± standard deviation.

Since huge differences were observed concerning growth in the amoebae plate assay we investigated three different yeast strains in more detail: the Baker's yeast, the bloodstream isolate RKI 07-0060, and the laboratory strain w303a/α (compare Figure 2A). To get first insights into the possible mechanisms underlying the differences observed in the amoeba plate test we analyzed the growth of the different strains in various liquid media (YPD, HL5, AXoM; Figure 3). In all three tested media the Baker's yeast showed very robust growth, whereas the laboratory strain w303a/α always showed the lowest OD_600_ value. The bloodstream isolate RKI 07-0060 showed intermediate growth between Baker's yeast and w303a/α (Figures 3A–C).

Using AXoM we next investigated the phagocytosis of the different yeast strains by the amoebae. Here, strain w303a/α showed the highest uptake rate and the Baker's yeast showed the significantly lowest uptake rate. Again, strain RKI 07-0060 showed an intermediate uptake rate between w303a/α and Baker's yeast (Figure 3D).

**Figure 4:**
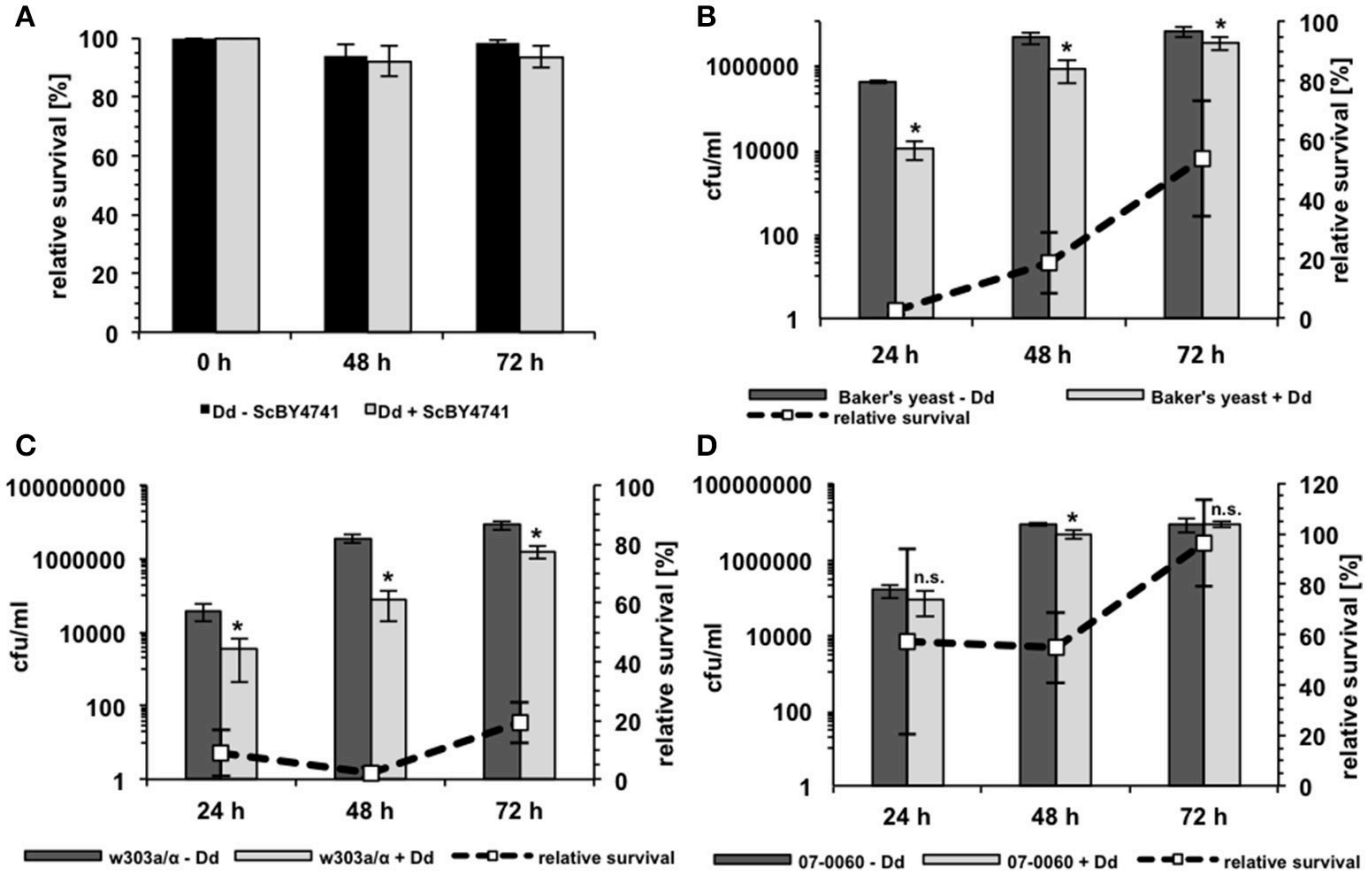
**Survival of *D. discoideum* and *S. cerevisiae* during co-incubation. (A)** Survival of *D. discoideum* AX2 (Dd) in SP-buffer with (gray bars) or without (black bars) *S. cerevisiae* BY4741 as revealed by trypan blue staining. No significant differences could be detected. **(B)** Survival of Baker's yeast with (light-gray bars) or without (dark-gray bars) *D. discoideum* AX2 in AXoM as measured by counting colony-forming units (cfu). **(C)** Survival of the laboratory *S. cerevisiae* strain w303a/α with (light-gray bars) or without (dark-gray bars) *D. discoideum* AX2 in AXoM as measured by counting cfu. **(D)** Survival of the *S. cerevisiae* bloodstream isolate RKI 07-0060 with (light-gray bars) or without (dark-gray bars) *D. discoideum* AX2 in AXoM as measured by counting cfu. The dashed lines in (**C,D)** represent the percentage survival rate of the yeast strain during co-incubation with *D. discoideum* as compared to the survival rate without amoebae (= 100%). All data are represented as mean ± standard deviation. ^*^significant reduction of cfu/ml compared to yeast cells without amoebae with *P* < 0.05. n.s., no significant difference.

The growth of the yeast during co-incubation with *D. discoideum* was further analyzed in 96-well plates in AXoM by counting colony forming units (cfu) at the indicated time points. Additionally, the survival of *D. discoideum* during co-incubation with yeast cells was monitored using the trypan-blue exclusion assay. Here, the cells were incubated in SP-buffer to check if the amoebae could potentially use the yeast cells as nutrients. As can be seen in Figure 4A, no significant difference could be observed in the survival of *D. discoideum* with or without yeast cells. This result was also independent of the *S. cerevisiae* strain used (data not shown). On the contrary, growth of the yeast strains during co-incubation with amoebae was strain-dependent (Figures 4B–D). Strain w303a/α showed the lowest number of cfu/ml, whereas strain RKI 07-0060 showed the highest number of cfu/ml at all tested time points. In this assay, Baker's yeast showed an intermediate number of cfu/ml compared to the other two tested strains. At later time points, cfu/ml of w303a/α also slightly increased. However, the numbers did not reach the values of the Baker's yeast or strain RKI 07-0060, probably due to slower growth of strain w303a/α in the AXoM used (compare Figure 3C).

The interaction of the three different yeast strains with *D. discoideum* was also followed using time-lapse microscopy for 4–5 h. As can be seen in the Movies M1–M3 (see also Supplementary Material for a detailed description), the amoebae rapidly took up *S. cerevisiae* w303a/α cells (Movie M1). In contrast, the phagocytosis of the patient isolate RKI 07-0060 was strongly reduced (Movie M2). The Baker's yeast even showed flocculation of cells (clumps in Movie M3) combined with a reduced uptake in submerged cultures.

Taken together, growth of *S. cerevisiae* on amoeba plates shows strain specific patterns that may be influenced by different fungal determinants such as the growth rate in different media, phagocytosis by the amoebae, and survival of the yeast during co-incubation.

### Host factors involved in the interaction between *D. discoideum* and fungi

**Figure 5:**
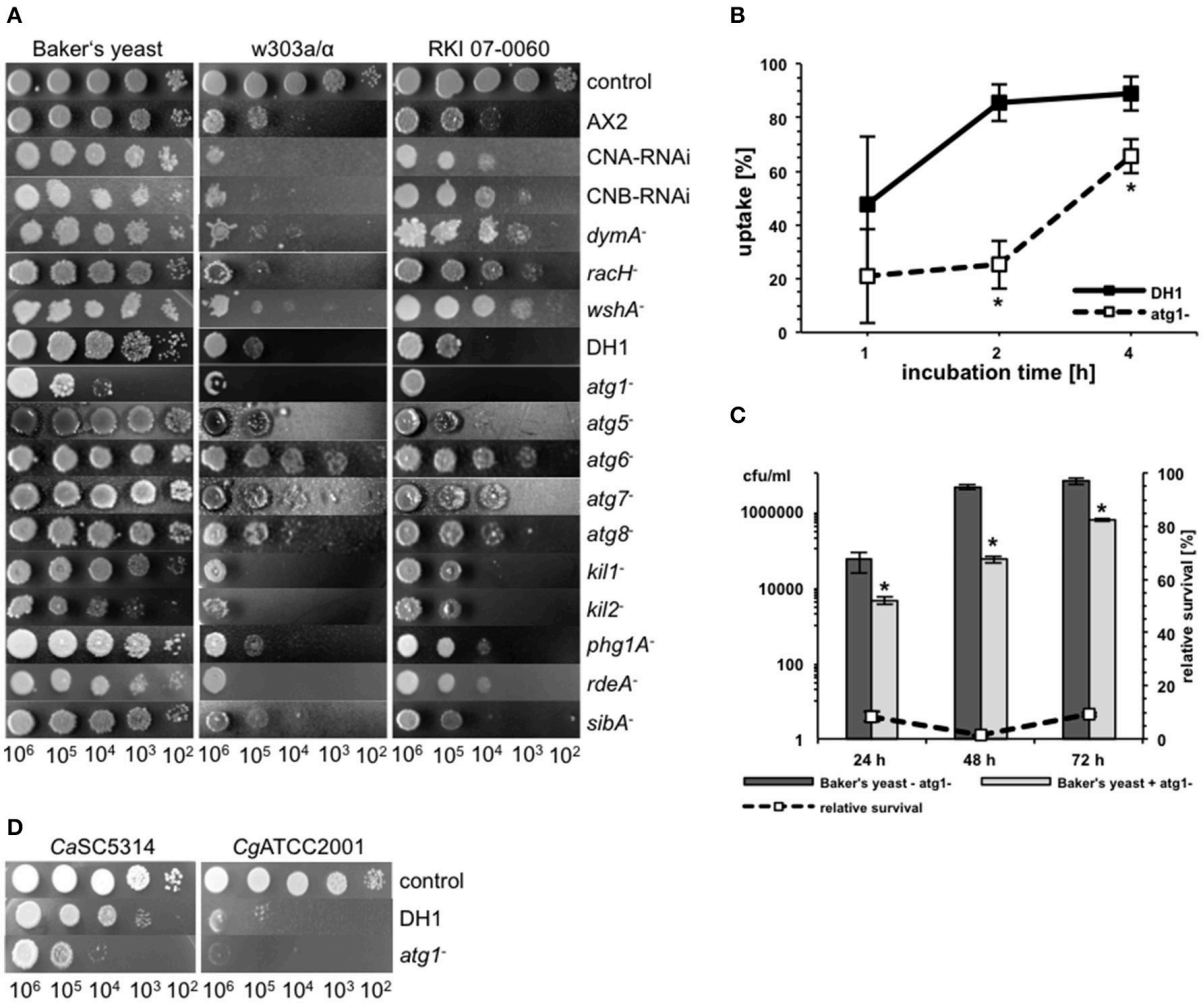
**Different host factors are involved in the interaction of *D. discodieum* with yeast cells**. **(A)** Amoebae plate test of the three *S. cerevisiae* strains Baker's yeast, w303a/α, and RKI 07-0060 in combination with different *D. discodieum* mutant cell lines and their parental strains AX2 and DH1. As a control, yeast cells were spotted on HL5-agar without amoebae. **(B)** Phagocytosis-rate of *S. cerevisiae* w303a/α with *D. discoideum atg1*^−^ mutant (dashed line) and its parental strain DH1 (solid line). Data are represented as mean ± standard deviation. ^*^significant difference between *atg1*^−^ and DH1 with *P* < 0.05. **(C)** Survival rate of Baker's yeast during co-incubation with (light-gray bars) or without (dark-gray bars) *atg1*^−^. The dashed line represents the percentage survival rate of the yeast strain during co-incubation with *D. discoideum* as compared to the survival rate without amoebae (= 100%). Data are represented as mean ± standard deviation. ^*^significant reduction of cfu/ml compared to yeast cells without *atg1*^−^ with *P* < 0.05. **(D)** Amoebae plate test of *D. discoideum atg1*^−^ and its parental strain DH1 with pathogenic *C. albicans* and *C. glabrata* strains.

In contrast to other amoebae like *Acanthamoeba castellanii, D. discoideum* is highly amenable to genetic manipulations. Accordingly, the central resource platform for Dictyostelid genomics dictyBase (Kreppel et al., 2004) hosts the Dicty Stock Center, where many mutant cell lines of *D. discoideum* are provided (Fey et al., 2013). We used this advantage of *D. discoideum* over other host models to investigate putative host factors involved in the interaction with yeast cells (Figure 5A). First, we investigated mutants, which have already been shown to be involved in the interaction with pathogenic bacteria. The strains used comprised mutations in genes involved in phago- or exocytosis (*racH*^−^, *wshA*^−^, *phg1A*^−^, *sibA*^−^) (Cornillon et al., 2000, 2006; Somesh et al., 2006; Carnell et al., 2011), genes involved in intracellular killing of bacteria (*kil1*^−^, *kil2*^−^) (Benghezal et al., 2006; Lelong et al., 2011), genes involved in the general stress response (CNA-RNAi, CNB-RNAi) (Boeckeler et al., 2006; Thewes et al., 2014), and genes involved in autophagy or phagosome maturation (*atg1*^−^, *atg5*^−^, *atg6*^−^, *atg7*^−^, *atg8*^−^, *dymA*^−^) (Wienke et al., 1999; Otto et al., 2003, 2004; Gopaldass et al., 2012). Additionally, we tested a mutant strain, which showed a precocious developmental cycle (*rdeA*^−^) (Chang et al., 1998).

As demonstrated in Figure 5A, mutations in most of the genes had no significant effect on the predation potential of *D. discoideum* toward yeast cells. However, some gene mutations resulted in slight to moderate impairment of the amoebal predation potential in a yeast-strain-dependent manner. For instance, the calcineurin RNAi mutants CNA-RNAi and CNB-RNAi, respectively, showed slight reduction in growth of the laboratory strain w303a, whereas the other two yeast strains were not affected in their growth. Similar results could be observed for *kil1*^−^ and *rdeA*^−^. For the *kil2*^−^ mutant growth of w303a and Baker's yeast was reduced. The most prominent mutations affecting growth of the different yeast strains were involved in autophagy of *D. discoideum*. Here, two types of effects could be observed: the *atg1*^−^ mutant impaired growth of all three yeast strains, whereas the *atg6*^−^ mutant promoted an increase of growth of all yeast strains. The weaker growth of the yeast during co-incubation with the *atg1*^−^ mutant seemed to be in contrast to earlier publications using pathogenic bacteria (Jia et al., 2009; Lampe et al., 2016). Therefore, we asked if the *atg1*^−^ mutant had defects in phagocytosis of yeast cells, if the growth inhibition rate is really higher than in the wild type, or if the mutant could also abolish the growth of pathogenic yeast. Concerning the uptake, Figure 5B shows that phagocytosis of w303a/α was significantly reduced after 2 and 4 h in the *atg1*^−^ mutant compared to the parental strain DH1. The yeast survival rate in co-incubation with *atg1*^−^ cells was lower than in wild type *D. discoideum* cells (Figure 5C) and *atg1*^−^ affected also growth of *C. albicans* and *C. glabrata* (Figure 5D).

Taken together, host factors involved in the intracellular killing of bacteria such as Kil1 and Kil2 are also relevant for the interaction with yeast cells, whereas factors involved in phago- and exocytosis or the general stress response are only slightly involved. Impairment of autophagy of the host plays a prominent role for the interaction with different effects: complete loss of autophagosome formation (*atg1*^−^) results in decreased growth of yeast cells whereas mutations in other autophagy genes (e.g., *atg6*^−^) results in increased growth of yeast cells.

### Fungal virulence factors determine interaction of *D*. *discoideum* with *C. albicans*

**Figure 6:**
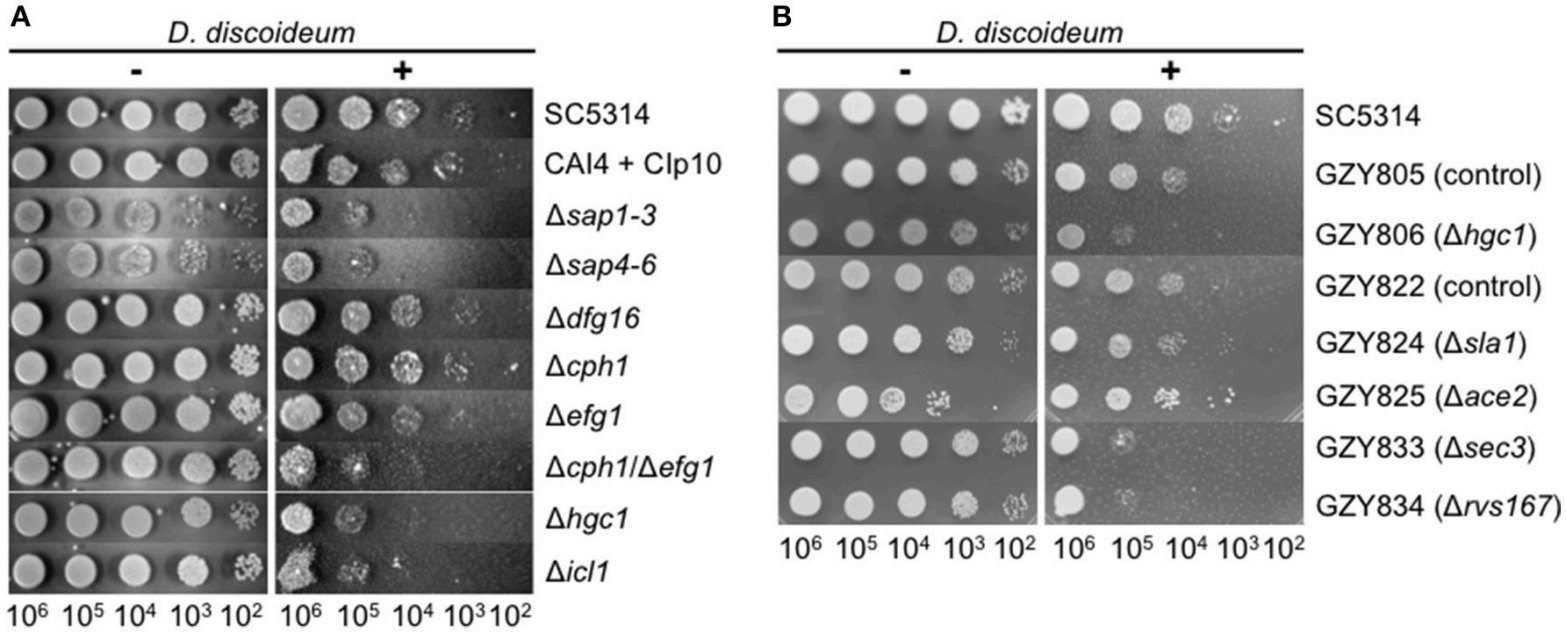
**Known virulence factors of *C. albicans* are involved in the interaction with *D. discoideum* AX2. (A)** Amoebae plate tests of different diploid *C. albicans* mutant strains and their parental strains SC5314 and CAI4 + CIp10, respectively. **(B)** Amoebae plate tests of different haploid *C. albicans* mutant strains and their parental strains GZY805 and GZY822, respectively.

Besides the investigation of the interaction of *D. dicoideum* with different non-pathogenic yeast strains we tested whether the amoebae could be used as a tool for identifying putative virulence factors of pathogenic yeast. Therefore, we investigated the role of known *C. albicans* virulence factors in the amoebae plate test. At first, we chose mutant strains with defects in hyphae formation (Δ*cph1*, Δ*dfg16*, Δ*efg1*, Δ*hgc1*, Δ*cph1*/Δ*efg1*) (Liu et al., 1994; Lo et al., 1997; Zheng et al., 2004; Thewes et al., 2007), mutants with defects in the expression of secreted aspartic proteases (Δ*sap1-3*, Δ*sap4-6*) (Sanglard et al., 1997; Kretschmar et al., 2002), as well as a mutant with defects in the glyoxylate cycle (Δ*icl1*) (Ramírez and Lorenz, 2007). Figure 6A reveals that the non-filamentous Δ*cph1*/Δ*efg1* double mutant, the non-filamentous Δ*hgc1* mutant, and the glyoxylate cycle mutant Δ*icl1* had reduced resistance toward predation by the amoebae. The Δ*efg1* mutant was only slightly affected and the Δ*cph1* and Δ*dfg16* mutants were not affected. The secreted aspartic protease mutants Δ*sap1-3* and Δ*sap4-6* also showed a growth reduction in association with amoebae compared to the control strains. However, these mutant strains also displayed a growth reduction on the AXoM plates without amoebae (Figure 6A, left panel). We confirmed that the mutants with defects in hyphae formation also formed no hyphae in contact with *D. discoideum* (Figure S3A).

Recently, stable haploid *C. albicans* strains have been described (Hickman et al., 2013). We wondered if the amoebae plate test could be used with those strains. Therefore, the resistance of the haploid *C. albicans* strains GZY805 and GZY822 toward predation by *D. dicoideum* was compared, and we found that both strains were slightly more susceptible compared to the “parental” diploid strain SC5314 (Figure 6B). Furthermore, we tested the available haploid mutant strains, which also have defects in hyphae formation. For the haploid Δ*hgc1* strain (GZY806), as well as for the haploid mutant strains Δ*sec3* (GZY833) and Δ*rvs167* (GZY834), we found a reduction in the resistance toward predation compared to the parental control strains GZY805 and GZY822, respectively (Figure 6B). For the haploid mutant Δ*sla1* (GZY824) no difference was seen compared to the control strain and Δ*ace2* (GZY825) showed a slight increase in the resistance toward predation. Investigating the ability of the haploid mutants to produce hyphae in contact with *D. discoideum* revealed that besides the haploid Δ*hgc1*, all other mutants still produced at least pseudohyphae (Figure S3B). Furthermore, the haploid status of the cells was confirmed by propidium iodide staining and subsequent flow cytometry (Figure S4).

Taken together, these results show that known virulence factors of *C. albicans* such as hyphae formation and a functional glyoxylate cycle also play a role during the interaction with amoebae.

### *C. albicans* forms hyphae in association with *D. discoideum* in a contact-independent manner

**Figure 7:**
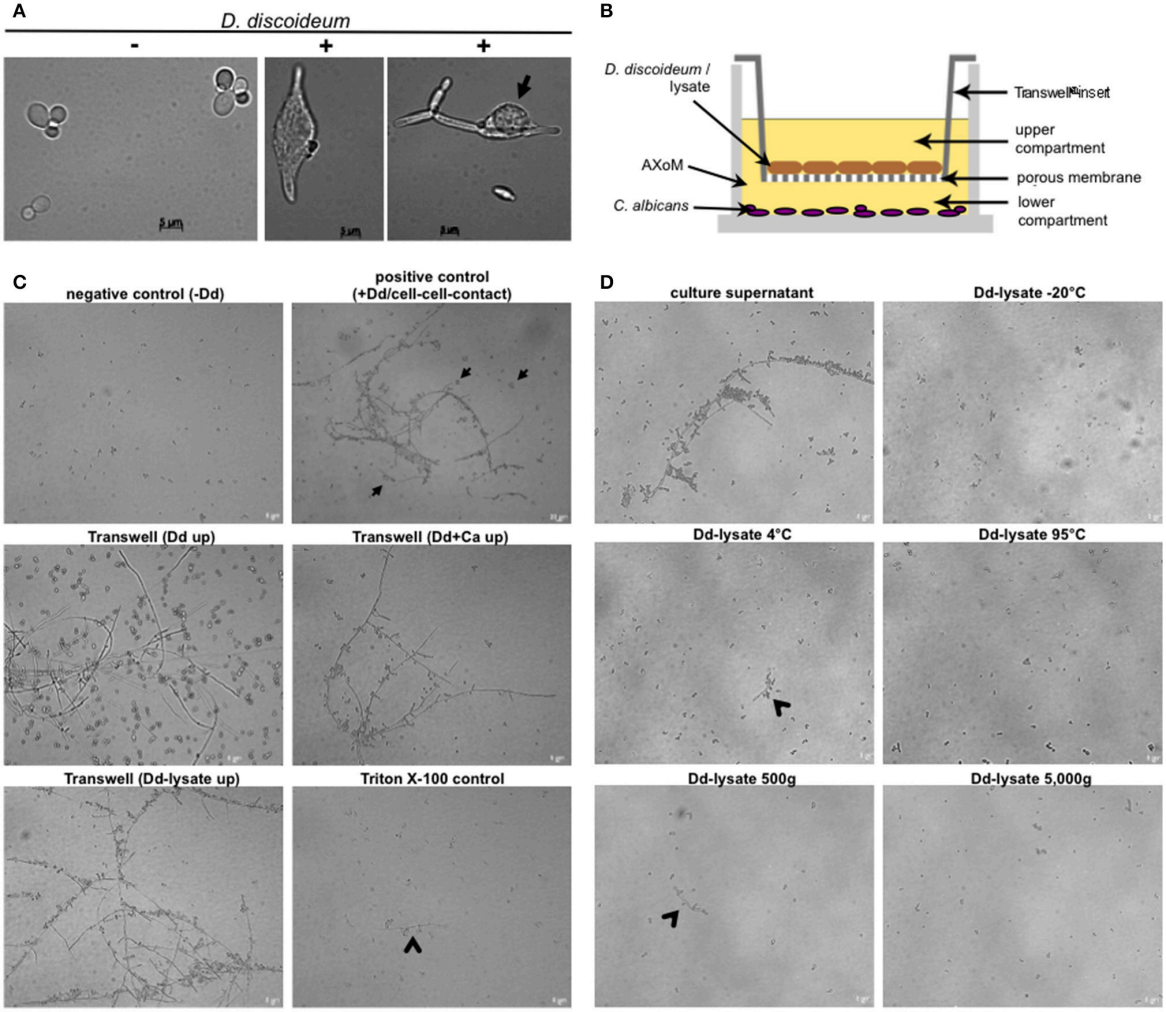
**Hyphae formation of *C. albicans* during co-incubation with *D. discoideum* is contact-independent. (A)** *C. albicans* SC5314 produces hyphae inside (middle panel) and outside (right panel) *D. discoideum* AX2. Without amoebae no hyphae formation could be detected (left panel). The arrow indicates *D. discoideum* in direct contact with *C. albicans* filaments. **(B)** Scheme of the Transwell®-assay. Cells were separated in AXoM by a porous membrane (pore size 0.04 μm). The upper compartment contained *D. discoideum* AX2 cells or cell lysate. The lower compartment always contained *C. albicans* SC5314. **(C)** Results of the Transwell®-assay. As a negative control *C. albicans* cells were incubated in AXoM without *D. discoideum*, as positive control *C. albicans* was incubated in AXoM with *D. discoideum* AX2 but without Transwell®-insert (cell-cell contact; arrows). Arrowheads indicate pseudohyphae formation of *C. albicans*. **(D)** Hyphae formation of *C. albicans* with differentially treated *D. discoideum* AX2 cell-lysates. Only untreated culture supernatant was able to induce hyphae formation. Arrowheads indicate pseudohyphae formation.

Although the temperature (22°C) is quite low and the media used did not contain any hyphae-inducing substances we observed in our assays that *C. albicans* SC5314 (and other strains as well) formed hyphae after phagocytosis or contact with *D. discoideum* (Figure 7A). To test if direct cell-cell contact between yeast and amoebae was necessary, both species were incubated in 24-well plates using Transwell® inserts, which resulted in separation of *C. albicans* from *D. discoideum* by a porous membrane (Figure 7B). As can be seen in Figure 7C direct cell-cell contact was not necessary for the hyphae production of *C. albicans*. Furthermore, lysed *D. discoideum* cells also induced hyphae formation of *C. albicans* (Figure 7C).

To get first insights in the putative nature of the hyphae-inducing compound of *D. discoideum* we treated cell lysates in different ways, such as incubation at different temperatures (−20, 4, 95°C) or differential centrifugation (500 g, 5000 g). Figure 7D shows that treatment of the *D. discoideum* lysates with very low (−20°C) or very high (95°C) temperatures completely abolished hyphae formation of *C. albicans*. This effect was also observed after centrifugation of the lysate at 5000 g. Incubation of the cell lysate at moderately low temperature (4°C) and moderate centrifugation (500 g) led to a partial reduction of hyphae formation (Figure 7D). Since *D. discoideum* secretes cyclic adenosine-3,′5′-monophosphate (cAMP) upon starvation (Du et al., 2015), and the cAMP-protein kinase pathway is a central pathway for hyphal initiation in *C. albicans* (Lu et al., 2014), we thought that secreted cAMP might trigger the hyphae formation of *C. albicans*. Therefore, we added different concentrations (10–100 nM) of cAMP to *C. albicans* in AXoM but found no hyphae formation (Figure S5).

Although the temperature at 22°C is quite low for hyphae production of *C. albicans, D. discoideum* secretes a yet unidentified diffusible factor that can trigger hyphae formation.

## Discussion

It has been proven in the last 15 years that the social amoeba *D. discoideum* can be used as a suitable host model to study interaction with pathogenic bacteria. However, research dealing with the interaction of *D. discoideum* with pathogenic fungi is still limited. Only a few studies using *Cryptococcus neoformans* or *Aspergillus fumigatus* have been conducted so far (Steenbergen et al., 2003; Chrisman et al., 2011; Hillmann et al., 2015; Mattern et al., 2015). On the other hand, yeast cells have been used for a long time to study phagocytosis of *D. discoideum* (Rivero and Maniak, 2006) but the impact of different yeast strains, or the fate of the yeast cells was usually not investigated. We present for the first time a comprehensive study of the interaction of *D. discoideum* with apathogenic and pathogenic yeast; a convenient method for co-cultivation to investigate fungal strains is introduced as well.

### Yeast factors playing a role for the interaction with *D. discoideum*

After having successfully adapted the amoebae plate test originally developed by Albers et al. (2005), we observed that different *S. cerevisiae* strains showed differential susceptibility toward predation by the amoebae. As plaque assays used to study the interaction of *D. discoideum* with bacteria (Froquet et al., 2009) failed to study the interaction with yeast, our first intention was that the size of the yeast particles or flocs of yeast cells might have an impact. However, testing several flocculating yeast strains overexpressing different *flo*-genes (Smukalla et al., 2008; Beauvais et al., 2009; Nonklang et al., 2009), no correlation was observed between flocculation and resistance toward predation. Flocculation of yeast cells is a reversible, calcium-dependent process (Verstrepen et al., 2003) and it is likely that *D. discoideum* can release attached cells out of flocs. For *S. cerevisiae* strain TH2-1B, which has a budding defect, it has been shown that *D. discoideum* is able to divide incompletely-budded yeast particles, allowing them to engulf the first encountered portion (Clarke, 2010). TH2-1B has deletions in the genes *MNN1* and *MNN2*, both encoding mannosyltransferases, and the mutant strain is altered in the structure of the cell wall mannoprotein, resulting in the budding defect (Clarke, 2010). Nevertheless, as TH2-1B was partially resistant toward predation in our assay, particle size might contribute at least partially to the observed resistance. The role of the particle size is further supported by the results obtained with non-axenic amoebal species. None of the non-axenic species was able to reduce the growth of the yeast cells. Recently it has been shown that only axenic *D. discoideum* strains are able to phagocytize bigger particles such as yeast cells, due to a mutation in *axeB*, a gene encoding neurofibromin that limits the size of macropinosomes (Bloomfield et al., 2015). This might be the reason why non-axenic strains were not able to phagocytize yeast cells. But it is not only the particle size that has an impact on the predation of yeast cells by *D. discoideum*. Other factors must play a role, since the “natural” Baker's and Brewer's yeast—as well as the bloodstream isolates—were resistant toward predation without having a bigger particle size compared to predation-susceptible laboratory strains. Different virulence-related traits have been described in *S. cerevisiae*, including growth at elevated temperatures and the ability to form pseudohyphae (McCusker et al., 1994), phenotypic switching (Clemons et al., 1996), or resistance to oxidative stress (Diezmann and Dietrich, 2009). We tested several phenotypic traits of the different yeast strains including growth at 42°C, resistance toward H_2_O_2_, or resistance toward cell surface perturbing agents such as SDS or congo red; we found no big differences between the tested strains that could have been correlated to the resistance against predation of the yeast by amoebae (data not shown). However, Baker's yeast and the bloodstream isolate RKI 07-0060 showed better growth in liquid culture as well as reduced phagocytosis rates compared to strain w303a/α. Therefore, general growth characteristics and the ability to prevent phagocytosis correlate to the resistance toward predation on our amoeba plate test. Time-lapse microscopy revealed that yeast strains might have evolved different strategies to face predation by amoebae. Whereas laboratory strains have probably lost the ability to resist predation, bloodstream isolates were obviously not efficiently recognized by *D. discoideum*, leading to reduced uptake. Further, flocculation of yeast cells, as can be seen for the Baker's yeast, was an effective strategy to avoid predation. However, flocculation as a tool to prevent phagocytosis seems to work only in submerged cultures in contact with amoebae. During co-incubation on solid substrates, as in the amoebae plate test, flocculation of yeast cells had only a minor impact and the Baker's yeast showed no increased flocculation when grown in YPD. The molecular basis for these differences will need to be determined in future studies, yet slight differences in cell wall composition of the different strains might be a reason for the observed differences concerning phagocytosis. For macrophages, several studies have shown that different cell wall components, especially mannan, are needed for the recognition of yeast cells by the phagocyte (Keppler-Ross et al., 2010; McKenzie et al., 2010; Lewis et al., 2012). However, further details of the interaction between yeast and macrophages are only poorly understood (Kong and Jabra-Rizk, 2015). In the future, *D. discoideum* might be a suitable host model to elucidate further details of yeast-phagocyte interaction. Additionally, the amoeba plate test—in combination with other methods to determine virulence-related traits—might be useful for risk assessment of *S. cerevisiae* strains.

### Growth of yeast cells is affected by different host factors

Since—in contrast to many other host models—*D. discoideum* is highly amenable to genetic manipulations, amoebal factors, which might be involved in the interaction with yeast cells, were investigated. At first, we concentrated on factors that have been described as playing a role in the interaction with bacteria. Whereas genes involved in phago-/exocytosis or phagosome maturation (*racH*^−^, *wshA*^−^, *phg1A*^−^, *sibA*^−^, *dymA*^−^) (Wienke et al., 1999; Cornillon et al., 2000, 2006; Somesh et al., 2006; Carnell et al., 2011) and genes involved in the general stress response (CNA-RNAi, CNB-RNAi) (Boeckeler et al., 2006; Thewes et al., 2014) were only slightly involved in the interaction with yeast cells, genes involved in intracellular killing of bacteria (*kil1*^−^, *kil2*^−^) (Benghezal et al., 2006; Lelong et al., 2011) and genes involved in autophagy (*atg1*^−^, *atg5*^−^, *atg6*^−^, *atg7*^−^, *atg8*^−^) (Otto et al., 2003, 2004; Gopaldass et al., 2012) had a larger impact during the interaction with different *S. cerevisiae* strains. The gene *kil1* encodes the only characterized sulfotransferase in *Dictyostelium* and mutation of this gene led to defects in intracellular killing of bacteria probably via a still unidentified sulphated factor (Le Coadic et al., 2013). This effect seems to be bacteria-specific, since increased predation of yeast cells by the *kil1*^−^ mutant was observed during the interaction with yeast. Similar results were obtained with the mutant *kil2*^−^. Kil2 is a putative magnesium pump involved in maintaining the phagosomal magnesium concentration optimal for proteolysis and efficient killing of *Klebsiella pneumoniae* (Lelong et al., 2011). Killing of other bacteria was not affected by the mutation of *kil2*. In our assay, similar to *kil1*^−^, the *kil2*^−^ strain showed increased predation of yeast cells. Both genes, *kil1* and *kil2*, therefore seem to have species-specific roles during intracellular killing of the prey. However, the underlying mechanisms are not fully understood: neither for bacteria nor for yeasts, and must be elucidated in future studies.

Concerning the autophagy mutants two opposite and counterintuitive phenotypes could be detected in the amoebae plate test: *atg1*^−^ cells showed higher predation of yeast cells, whereas *atg6*^−^ cells showed lower predation of yeast cells. It is known that mutations in different autophagy-related genes led to differences in the phenotypical outcome with *atg1*^−^ always showing the most severe defects compared to other *atg*-mutants (Otto et al., 2004). Atg1 is required for the initiation of the autophagosome formation. Loss of *atg1* completely blocks autophagosome formation, whereas loss of other components of the autophagic machinery can still result in production of very small autophagosomes (Abeliovich et al., 2000). However, we would have suspected that impairment of autophagy results in an increased growth of yeast cells, since autophagy has been connected to cellular immunity (Deretic et al., 2013; Winchell et al., 2016). This seems to be partially true for *D. discoideum atg6*^−^ but for the *atg1*^−^ mutant the opposite effect was observed, which is in contrast to studies using pathogenic bacteria (Jia et al., 2009; Lampe et al., 2016). The reason for this discrepancy is currently not known, but since the *atg1*^−^ mutant is not able to produce autophagosomes at all, the cells are unable to recycle cellular components to gain nutrients and the cells might therefore be “hungrier,” allowing efficient degradation of yeast cells. Another possible explanation would be that yeast cells use the autophagic machinery to escape from phagocytes, as has been shown for mycobacteria (Gerstenmaier et al., 2015). Non-lytic expulsion from macrophages has also been observed for yeast cells (Ma et al., 2006; Bain et al., 2012). It might be possible that yeast cells are “locked” in *atg1*^−^ mutants, giving the amoeba the ability to efficiently kill the yeasts. Yeast cell uptake seems to have a minor impact in the *atg1*^−^ mutant, as the reduced phagocytosis rate does not correlate with the increased growth inhibition. Future studies will have to dissect the exact role of Atg1 for phagocytosis, intracellular killing, and non-lytic expulsion of yeast cells in *D. discoideum*.

In general, the impact of autophagy for the interaction with fungi is controversially discussed and depends on the investigated fungus, the host, and the *atg*-gene (Lenz et al., 2011; Qin et al., 2011; Nicola et al., 2012; Rosentul et al., 2014; Smeekens et al., 2014; Kanayama et al., 2015; Akoumianaki et al., 2016). Investigating different *atg*-mutants in the same host with different fungal species, our study shows that the impact of autophagy seems to be related to the mutated *atg*-gene. This again reflects the power of *D. discoideum* as a suitable host model to investigate the interaction with different yeast species.

At this point, it should be mentioned that *atg1*^−^ mutants cannot develop properly and arrest in their developmental cycle as loose mounds (Otto et al., 2004). This phenotype might have an impact in our amoebae plate test (longer persistence as single amoebae = higher predation rate). However, the *phg1A*^−^ mutant has a similar developmental defect compared to the *atg1*^−^ mutant (Benghezal et al., 2003) and showed no increased predation of yeast cells in our assay. Additionally, we tested a “rapid development” mutant (*rdeA*^−^) (Chang et al., 1998) and found a rather increased predation than decreased predation of yeast cells. These results indicate that the persistence as amoebal cell *per se* only had minor impact on the interaction with yeast cells in the amoebae plate test.

### Known virulence factors of *C. albicans* are required for the interaction with *D. discoideum*

In order to validate the power of *D. discoideum* as a host model system for pathogenic yeast we investigated the role of known *C. albicans* virulence factors during co-incubation with the amoebae. Mutant strains with defects in hyphae formation (Δ*cph1*, Δ*dfg16*, Δ*efg1*) had no or only little effect on the interaction with *D. discoideum*, although all three mutants formed no hyphae (Δ*dfg16*) or merely short pseudohyphae (Δ*cph1*, Δ*efg1*) in contact with amoebae. Only the Δ*hgc1* mutant and the Δ*cph1*/Δ*efg1* double mutant, both locked in the yeast form (Lo et al., 1997; Zheng et al., 2004), showed reduced resistance toward predation by the amoebae. This correlates with earlier results showing that, beside morphogenesis defects, the Δ*hgc1* and Δ*cph1*/Δ*efg1* mutants also have a markedly reduced virulence (Δ*hgc1*) (Zheng et al., 2004) or are completely avirulent (Δ*cph1*/Δ*efg1*) (Lo et al., 1997) in a mouse model. The observed phenotypes of the Δ*cph1* and Δ*efg1* single mutants also are in accordance with a previous study investigating the interaction of *C. albicans* with human blood (Fradin et al., 2005). This study revealed that the Δ*cph1* mutant was not more susceptible toward predation by monocytes than the wild type. Further, the Δ*efg1* mutant showed a slight decrease in its survival rate during incubation with monocytes, which is comparable to the slightly reduced growth of the mutant in the amoebae plate test. The putative pH-sensor Dfg16 is involved in the correct pH-sensing under alkaline conditions (Barwell et al., 2005; Thewes et al., 2007). Although the Δ*dfg16* mutant showed no increased susceptibility toward predation by amoebae, it was not able to form hyphae during co-incubation with *D. discoideum*. The pH value of the AXoM medium used is around 6.7, indicating that the pH alone is not responsible for the filamentation defect of Δ*dfg16*. Similar results were obtained using the cell culture medium M199 adjusted to different pH values. Here, the wild type produced hyphae in the range of pH 5-8, whereas Δ*dfg16* produced no hyphae at all at pH 8 and had strong filamentation defects at pH 7 and below (Thewes et al., 2007 and unpublished results). The results of the interaction of *D. discoideum* with different mutants defective in hyphae formation imply that—beside morphogenesis—other factors regulated via Hgc1, Cph1, and/or Efg1 are crucial for the survival of *C. albicans* during co-incubation with amoebae. The significance of hyphae formation for the resistance toward predation is underlined by the observed hyphae formation of *C. albicans* even without direct contact to *D. discoideum*. This is in contrast to *C. neoformans*, where direct cell-cell contact between fungus and amoebae was necessary for capsular enlargement (Chrisman et al., 2011). These results led to the assumption that *D. discoideum* secretes a factor which triggers hyphae formation in *C. albicans*. This factor is not cAMP, which is secreted by starving *D. discoideum* cells (Du et al., 2015) and important for hyphal initiation in *C. albicans* (Lu et al., 2014). The nature of this heat- and cold-sensitive factor has to be determined in future studies.

Another protein involved in the interaction between *D. discoideum* and *C. albicans* is the isocitrate lyase Icl1, representing one of the key components of the glyoxylate cycle. For *S. cerevisiae* and *C. albicans* it has been shown that the expression of *ICL1* is up-regulated during co-incubation with macrophages (Lorenz and Fink, 2001; Lorenz et al., 2004). Accordingly, the *C. albicans* Δ*icl1* mutant is less virulent in mice (Lorenz and Fink, 2001; Ramírez and Lorenz, 2007). In our amoebae plate test the Δ*icl1* mutant also showed a reduced resistance toward predation by the amoebae. This demonstrates the significance of the glyoxylate cycle in microbial pathogenesis, especially for the interaction with phagocytic cells.

*C. albicans* was for a long time thought to be an obligate diploid yeast, but recently it was shown that it is possible to construct stable haploid strains of *C. albicans*, facilitating the molecular and genetic analysis of this important fungal pathogen (Hickman et al., 2013). We investigated whether *D. discoideum* could be used in combination with haploid *C. albicans* strains. In contrast to a mouse model of systemic candidiasis, in which the haploid *C. albicans* strain tested was avirulent (Hickman et al., 2013), virulence of haploid strains was only slightly reduced in our amoebae plate test in comparison to the highly virulent diploid strain SC5314. Additionally, haploid mutant strains like GZY806 (Δ*hgc1*) showed comparable phenotypes to its diploid counterpart. Other haploid mutant strains like GZY824 (Δ*sla1*) or GZY825 (Δ*ace2*) showed no reduced virulence (GZY824) or were even increased in virulence (GZY825). Sla1 is involved in hyphal growth by regulating actin patch dynamics and Δ*sla1* mutants show a defective hyphal development (Reijnst et al., 2010; Zeng et al., 2012). In association with *D. discoideum* the haploid Δ*sla1* mutant was still able to form (pseudo-) hyphae, which might contribute to the resistance toward predation. For the transcription factor Ace2 it has been shown that it is has different roles in filament formation depending on conditions (Kelly et al., 2004; Mulhern et al., 2006). However, under most tested conditions the Δ*ace2* mutant strain has shown hyperfilamentation, even under non-inducing conditions (Kelly et al., 2004). We also observed hyperfilamentation for the haploid Δ*ace2* strain GZY825. This might contribute to the slightly increased resistance of this strain toward predation by *D. discoideum*. Further, haploid mutant strains, for which the diploid counterparts show abnormal hyphal morphogenesis, such as the exocyst subunit Sec3 (Δ*sec3*) (Li et al., 2007) or the plasma membrane protein Rvs167 (Δ*rvs167*) (Douglas et al., 2009), were able to form filaments in contact with *D. discoideum*, but were more susceptible toward predation. At least for the haploid Δ*rvs167*, this result is in accordance with the reduced virulence of its diploid counterpart (Douglas et al., 2009). The haploid *C. albicans* mutant strains again demonstrate that hyphae formation alone is not always sufficient to mediate resistance toward predation by phagocytic cells.

The results obtained with pathogenic *C. albicans* strains emphasize the power of *D. discoideum* as a host model system. Although *D. discoideum* is no natural host for *C. albicans*, the investigation of the interaction of *C. albicans* with *D. discoideum* might result in the identification of fundamental and evolutionary conserved fungal as well as host factors important for the interaction.

## Conclusions

The concept of amoebae as training grounds for intracellular bacterial pathogens is well established. The fact that amoebae can also be used to study the interaction with fungal pathogens has been shown for different fungi (Steenbergen et al., 2001, 2003, 2004; Chrisman et al., 2010; Araujo Gde et al., 2012; Magditch et al., 2012; Hillmann et al., 2015; Madu et al., 2015). Most of these studies used *A. castellanii* as the amoebal host. However, the advantages of *D. discoideum* as a host model are evident: (i) *D. discoideum* is easy to cultivate and highly amenable to genetic manipulations (Fey et al., 2007; Gaudet et al., 2007), (ii) *D. discoideum* has a fully sequenced genome (Eichinger et al., 2005), and (iii) a central resource platform for genome information—as well as a stock center for plasmids, mutant strains, etc.—is available (Kreppel et al., 2004; Fey et al., 2013).

We now introduce *D. discoideum* as a suitable host model to study the interaction with apathogenic and pathogenic yeast. Our modified methods allow the use of *D. discoideum* for risk assessment of various yeast strains, as well as for the investigation of host-pathogen interactions on a cellular level. Limiting factors of our methods are the relative low temperature (22°C) during amoebae-fungus interaction and the fact that yeasts are not the preferred food source for *D. discoideum*, leading to overgrowth of the amoebae by non-phagocytized and non-killed yeast during longer co-incubation. However, the details of the interaction between *C. albicans* and phagocytic cells like macrophages are only poorly understood (Kong and Jabra-Rizk, 2015). Investigating the interaction of pathogenic yeast with the genetically highly amenable *D. discoideum* amoebae might help to elucidate new (evolutionarily conserved) aspects of fungus-phagocyte interaction. In combination with haploid *C. albicans* strains the amoeba might be useful to identify new virulence factors in large-scale mutagenesis methods.

## Author contributions

BK and SS carried out amoeba plate tests, growth assays, and the analysis of hyphae formation; CS and ED did amoeba plate tests and phagocytosis and killing assays, CS additionally worked with haploid *C. albicans* strains (including flow cytometry); JT did amoeba plate tests, the analysis of flocculation, and plaque assays; AP analyzed non-axenic Dictyostelia; VR and ST conceived the experiments and wrote the manuscript. All authors read and approved the final version of the manuscript.

### Conflict of interest statement

The authors declare that the research was conducted in the absence of any commercial or financial relationships that could be construed as a potential conflict of interest.
