# Supplementary material for: Dictyostelium discoideum as a Novel Host System to Study the Interaction between Phagocytes and Yeasts
Source: Front Microbiol. 2016 Oct 21;7:1665. doi: 10.3389/fmicb.2016.01665 (PMC5073093; doi:10.3389/fmicb.2016.01665)
Supplement: Supplementary file 4 [file DataSheet1.PDF]

Supplementary material

# *Dictyostelium discoideum* as a novel host system to study the interaction between phagocytes and yeasts

Barbara Koller, Christin Schramm, Susann Siebert, János Triebel, Eric Deland, Anna Maria Pfefferkorn, Volker Rickerts, and Sascha Thewes.

## Plaque-assay

To test whether *D. discoideum* was able to produce plaques on lawns of yeast we spread *S. cerevisiae* w303a/α on different media. To reduce the growth oft he yeast cells we used media with reduced amount of ingredients: 1/3 SM (3.3 g/l glucose; 3.3 g/l Bacto-peptone; 0.33 g/l yeast extract; 0.33 g/l $MgSO_4$ x 7 $H_2O$; 0.73 g/l $KH_2PO_4$; 0.43 g/l $K_2HPO_4$ x 3 $H_2O$; 2 % agar) and 1/3 and 1/4 YPD, respectively. Directly after spreading of the yeast, *D. discoideum* grown to mid-log phase in HL5 were spotted (5 µl) and plates were incubated at 22 °C. Appearance of plaques was monitored after 24 h and 48 h. As can be seen in figure S1, plaques were produced by *D. discoideum*. However, the plaques were not increasing in size over time. Spotting *D. discoideum* on already grown yeast lawns produced no plaques (not shown).

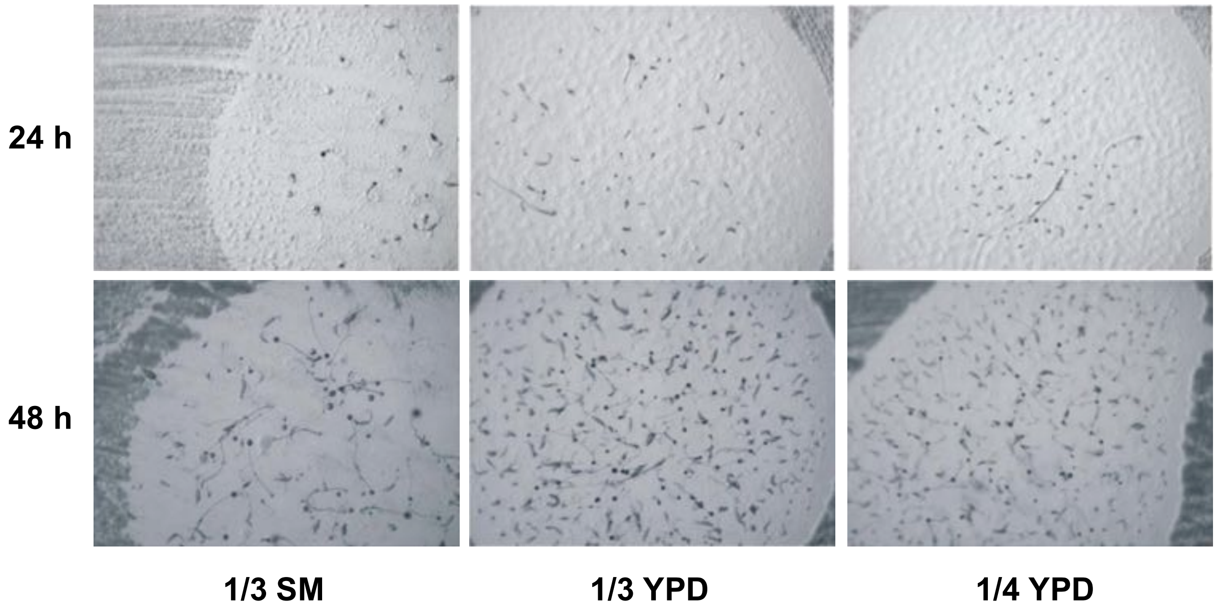


**FIGURE S1 | Plaque-assay.** *D. discoideum* cells were spotted directly after spreading of the *S. cerevisiae* w303a/α cells on different media. Plates were incubated at 22 °C.

## Yeast flocculation

We tested different yeast strains in the amoebae plate test. To investigate if flocculation of yeast might have an impact on the resistance towards predation by *D. discoideum*, several flocculating strains overexpressing *flo*-genes were tested. For validation of the flocculation-phenotype, yeast strains were grown in YPD over night at 30 °C and transferred in test tubes. The cell suspensions were mixed thoroughly and the tubes were left on the laboratory bench for 30 min. Only flocculating yeast strains showed strong sedimentation of yeast flocs (Fig.

S2). The yeast strain overexpressing *flo1* (*flo1*-OE) showed reduced sedimentation compared to the other *flo*-genes.

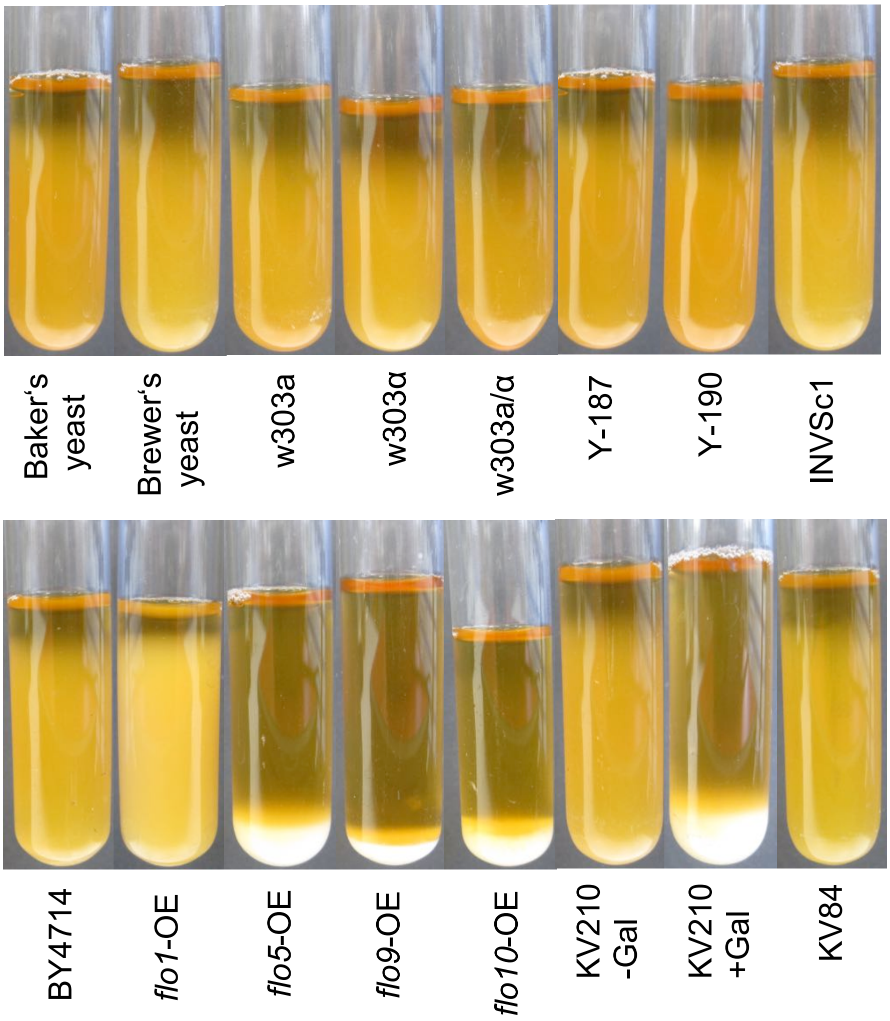


**FIGURE S2 | Flocculation test.** Yeast cells grown over night in YPD were mixed and left on the bench for 30 min. Flocculating strains showed sedimentation of yeast flocs with simultaneous clearing of the medium.

## Hyphae formation of *C. albicans* mutants

The ability to form hyphae is crucial for the interaction of *C. albicans* with host cells. We tested in our amoebae plate test different mutants including those who show defects in hyphae formation. To test if this defects also occur during co-incubation with *D. discoideum*, amoebae grown in HL5 were washed with SP-buffer, transferred to 24well plates, and *C. albicans* mutant cells and their parental strains were added. As a control *C. albicans* cells were incubated in AXoM without amoebae. Wild type cells as well as the Δ*sap1-3*, Δ*sap4-6*, and Δ*icl1* were able to produce hyphae in contact with *D. discoideum* (Fig. S3A). The mutant strains Δ*cph1* and Δ*efg1* only produced short pseudohyphae. All other tested mutant strains (Δ*dfg16*, Δ*cph1*/Δ*efg1*, Δ*hgc1*) showed no hyphae formation.
Similar results were observed for haploid *C. albicans* strains (Fig. S3B). Here, except for the haploid Δ*hgc1* mutant (GZY806), all strains were able to produce pseudohyphae or true hyphae in contact with *D. discoideum*.

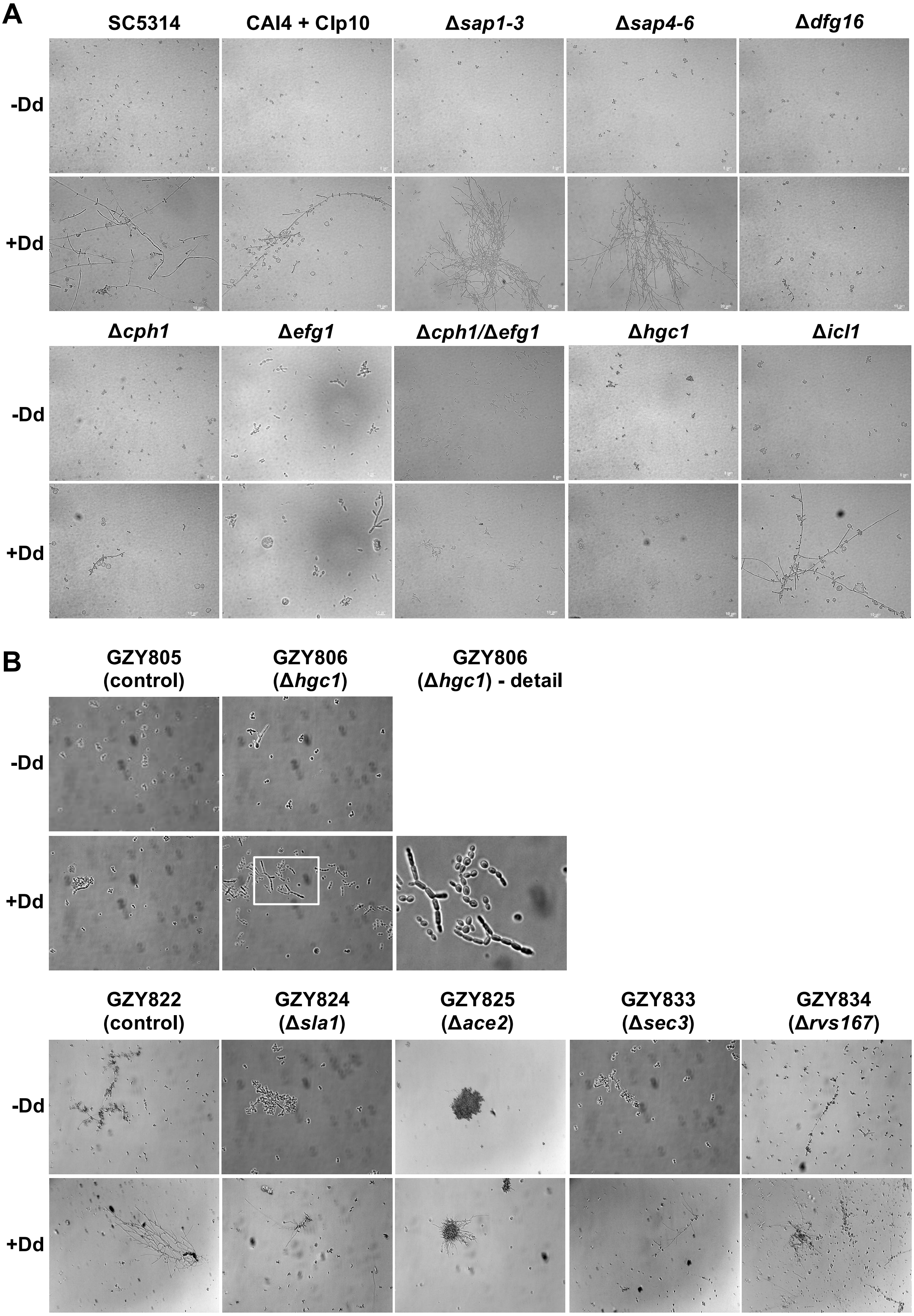


**FIGURE S3 | (A)** Hyphae formation of different diploid *C. albicans* wild type and mutant strains in contact with *D. discoideum*. **(B)** Hyphae formation of different haploid *C. albicans* strains in contact with *D. discoideum*. For strain GZY806 in contact with *D. discoideum* a detailed picture (white rectangle) is additionally shown aside. Strain GZY806 is not able to produce hyphae. Pictures were taken after 96 h.

## Propidium iodide staining and flow cytometry

To verify that the haploid *C. albicans* strains are still haploid, yeast cells were stained with propidium iodide and analyzed by flow cytometry. Figure S4 shows that all tested strains are haploid as compared to the diploid control strain SC5314. We were not able to analyze strain GZY825 (Δ*ace2*) in the flow cytometer due to its hyper-filamentous phenotype (compare figure S3).

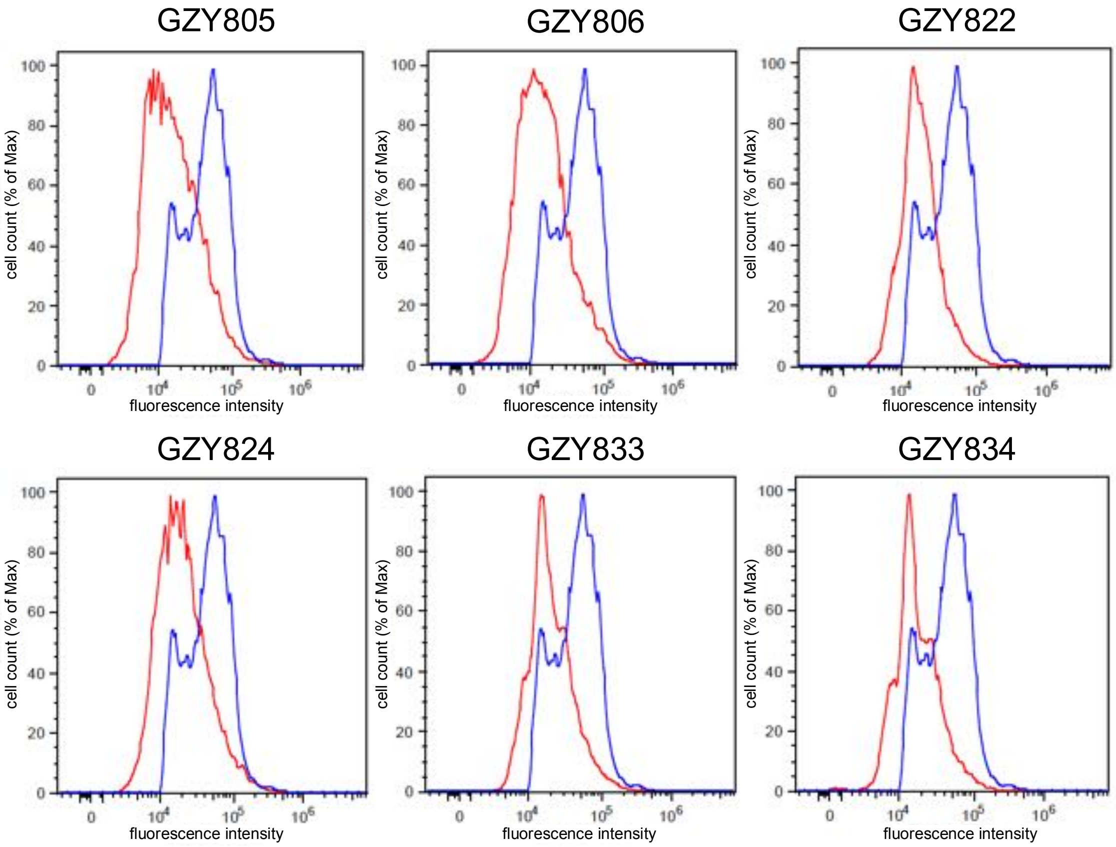


**FIGURE S4 | Flow cytometry of haploid and diploid *C. albicans* strains.** The red lines represent the indicated haploid *C. albicans* strain whereas the blue line always represents the diploid control strain SC5314.

## Extracellular cAMP does not induce hyphae formation

As *D. discoideum* secrets cAMP upon starvation and as (intracellular) cAMP is important for hyphae formation in *C. albicans* we tested the possibility that the diffusible factor, which induces hyphae formation of *C. albicans* during co-incubation with *D. discoideum*, might be cAMP. We added different concentrations of cAMP ranging from 5 nM to 100 nM to *C. albicans* SC5314. At all concentrations no hyphae formation was observed (Fig. S5). Sporadic, pseudohyphae were detected after cAMP treatment. This indicates that the diffusible factor responsible for hyphae formation of *C. albicans* is not (extracellular) cAMP.

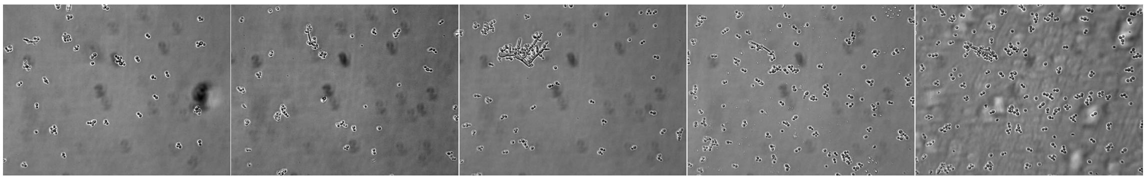

| 0 nM | 5 nM | 10 nM | 50 nM | 100 nM |
|---|---|---|---|---|

**FIGURE S5 | Treatment of *C. albicans* SC5314 with different concentrations of cAMP.** None of the chosen cAMP-concentrations induced hyphae formation.

## Supplementary movies:

The movies were generated according to the description in material and methods. After mixing of the cells, the wells were microscopically analyzed for suitable regions to take the pictures.

**Movie M1:**
Movie M1 shows the interaction of *D. discoideum* AX2 with the laboratory *S. cerevisiae* strain w303a/α. Yeast cells were taken up by the amoebae rapidly. At the end of the movie almost all free yeast cells were phagocytized. Some of the amoebae showed aggregation at the end of the video indicating putative starvation of the cells.

**Movie M2:**
Movie M2 shows the interaction of *D. discoideum* AX2 with the bloodstream *S. cerevisiae* isolate RKI 07-0060. Amoebae only hardly took up yeast cells. Over time, the number of yeast cells increased indicating growth of the yeast.

**Movie M3:**
Movie M3 shows the interaction of *D. discoideum* AX2 with the *S. cerevisiae* strain “Baker’s yeast”. As in video M2 yeast cells were only hardly taken up by the amoebae. At the end of the movie yeast cells and amoebae started to form clumps indicating putative flocculation of yeast cells as well as putative starvation of the amoebae.
